# Phosphoglycolate phosphatase homologs act as glycerol-3-phosphate phosphatase to control stress and healthspan in *C. elegans*

**DOI:** 10.1038/s41467-021-27803-6

**Published:** 2022-01-11

**Authors:** Elite Possik, Clémence Schmitt, Anfal Al-Mass, Ying Bai, Laurence Côté, Johanne Morin, Heidi Erb, Abel Oppong, Wahab Kahloan, J. Alex Parker, S. R. Murthy Madiraju, Marc Prentki

**Affiliations:** 1 Department of Nutrition, Université de Montréal, Montreal Diabetes Research Center, CRCHUM, Montréal, Canada; 2 Department of Biochemistry and Molecular Medicine, Montreal Diabetes Research Center, CRCHUM, Montréal, Canada; 3 Department of Neurosciences, CRCHUM, Montréal, Canada

**Keywords:** Enzyme mechanisms, Metabolomics

## Abstract

Metabolic stress due to nutrient excess and lipid accumulation is at the root of many age-associated disorders and the identification of therapeutic targets that mimic the beneficial effects of calorie restriction has clinical importance. Here, using *C. elegans* as a model organism, we study the roles of a recently discovered enzyme at the heart of metabolism in mammalian cells, glycerol-3-phosphate phosphatase (G3PP) (gene name *Pgp*) that hydrolyzes glucose-derived glycerol-3-phosphate to glycerol. We identify three *Pgp* homologues in *C. elegans* (*pgph*) and demonstrate in vivo that their protein products have G3PP activity, essential for glycerol synthesis. We demonstrate that PGPH/G3PP regulates the adaptation to various stresses, in particular hyperosmolarity and glucotoxicity. Enhanced G3PP activity reduces fat accumulation, promotes healthy aging and acts as a calorie restriction mimetic at normal food intake without altering fertility. Thus, PGP/G3PP can be considered as a target for age-related metabolic disorders.

## Abstract (web summary)

Glycerol-3-phosphate phosphatase is a recently discovered enzyme at the heart of metabolism. Here, the authors used *C. elegans* and showed that its activation promotes stress resistance, healthy aging and acts as a calorie restriction mimetic at normal food intake without altering fertility.

## Introduction

Glycerolipid/free fatty acid (GL/FFA) cycle links lipid and glucose metabolism and its dysregulation is associated with metabolic and age-related diseases^1,2^. This cycle, consisting of its lipogenesis and lipolysis arms, generates signaling molecules and promotes thermogenesis. During lipogenesis, glucose-derived glycerol-3-phosphate (Gro3P) is sequentially esterified with fatty acyl-CoAs to form triglycerides (TG). Upon limited nutrient supply, stored TG is hydrolyzed to glycerol and FFA^1,2^.

In biochemistry textbooks, lipolysis in mammals was believed to be the only source of glycerol, since they were thought to lack enzyme(s) converting Gro3P to glycerol. We have discovered a Gro3P phosphatase (G3PP) enzyme capable of hydrolyzing Gro3P to glycerol in mammalian cells^3^. Gro3P is a central metabolite at the crossroads of fat and carbohydrate metabolism. G3PP suppression in hepatocytes and pancreatic ß-cells increased lipid synthesis, reduced FFA oxidation and oxygen consumption, and lowered ATP production. Our findings implicated G3PP in the control of glycolysis, gluconeogenesis, cellular redox, and energy production when glucose is elevated^3^. However, the pathophysiological roles of G3PP in vivo are largely unknown.

Gro3P hydrolysis is a newly identified function of the previously described enzyme phosphoglycolate phosphatase (gene: *Pgp*)^4^ that belongs to the superfamily of haloacid dehalogenases (HAD)-like hydrolases. PGP also shows phosphatase activity in vitro towards several substrates besides Gro3P^3,5,6^, including 2-phosphoglycolate (2-PG) produced during DNA repair^7^, glycolysis-derived toxic side products such as 2-phospholactate and 4-phosphoerythronate^8^, and very low activity towards phosphotyrosine residues and nucleotides^3,9^. However, the physiological relevance of these activities remains to be defined in vivo, as the cellular concentrations of these metabolites are very low under normal culture conditions^5^.

Gro3P is a key intermediate of glycolysis, glycerolipid biosynthesis, gluconeogenesis, and the Gro3P redox shuttle. In conditions of excess glucose, Gro3P is redirected towards lipogenesis and the Gro3P shuttle, leading to fat deposition and metabolic stress/ ROS production, respectively, contributing to tissue damage and premature aging^10–14^. In contrast, dietary restriction is associated with reduced metabolism and it delays disease onset and extends lifespan in invertebrate^15–22^, vertebrate organisms^23–25^, and humans^26^. However, outstanding drawbacks of calorie restriction are that a substantial reduction in food intake is not applicable to humans and causes a major reduction in fertility. Thus, there is an outstanding interest in identifying pathways that could mimic calorie restriction without its associated caveats. The fact that G3PP hydrolyzes glucose-derived Gro3P to glycerol, a much less toxic molecule when produced in excess^27^, implies that this pathway might protect organisms from glucotoxicity and nutri-stress^28^. In addition, removal of glucose carbons as glycerol, that exit the cell through aquaglyceroporins, could mimic calorie restriction by reducing the availability of glucose for oxidation and lipogenesis. However, these candidate beneficial roles of G3PP have not been investigated in vivo.

Other than its metabolic functions, glycerol is an essential protective organic osmolyte produced to adapt high salinity environments^29,30^. In *Caenorhabditis elegans* (*C. elegans)*, exposure to hyperosmotic stress causes a rapid glycerol accumulation and is accompanied by the upregulation of mRNA levels of glycerol-3-phosphate dehydrogenase enzymes, *gpdh-1* and *gpdh-2* and glycogenolysis^31–35^. The roles of *gpdh-1* and *gpdh-2* enzymes have been examined in regard with hyperosmotic stress in *C. elegans*^31,32,36,37^. However, how Gro3P leads to glycerol synthesis downstream salt-driven glycogenolysis, and whether glycerol could be rapidly formed by an overlooked G3PP that mediates the hydrolysis of Gro3P in *C*. *elegan*s, is unknown.

To identify G3PP functions in vivo, we used *C*. *elegans* as a model system and studied many complex processes, particularly the response to various stresses and aging. Here, we show that *C. elegans* harbors three homologs of mammalian G3PP that we named phosphoglycolate phosphatase homologs (now listed as PGPH; WormBase). We demonstrate that the worm PGPH acts as a Gro3P phosphatase in vivo and that G3PP is involved in the response to various stresses, including excess glucose and hyperosmotic stress, as well as healthy aging. Also, we show that enhanced PGPH/G3PP activity reduces fat accumulation and in part mimics the beneficial effects of dietary restriction and promotes healthy aging, particularly in conditions of excess glucose, without adversely affecting the apparent food intake (pharyngeal pumping) or reproduction.

## Results

### The worm genome harbors three homologs of mammalian PGP/G3PP with glycerol-3-phosphate phosphatase function

**Fig. 1:**
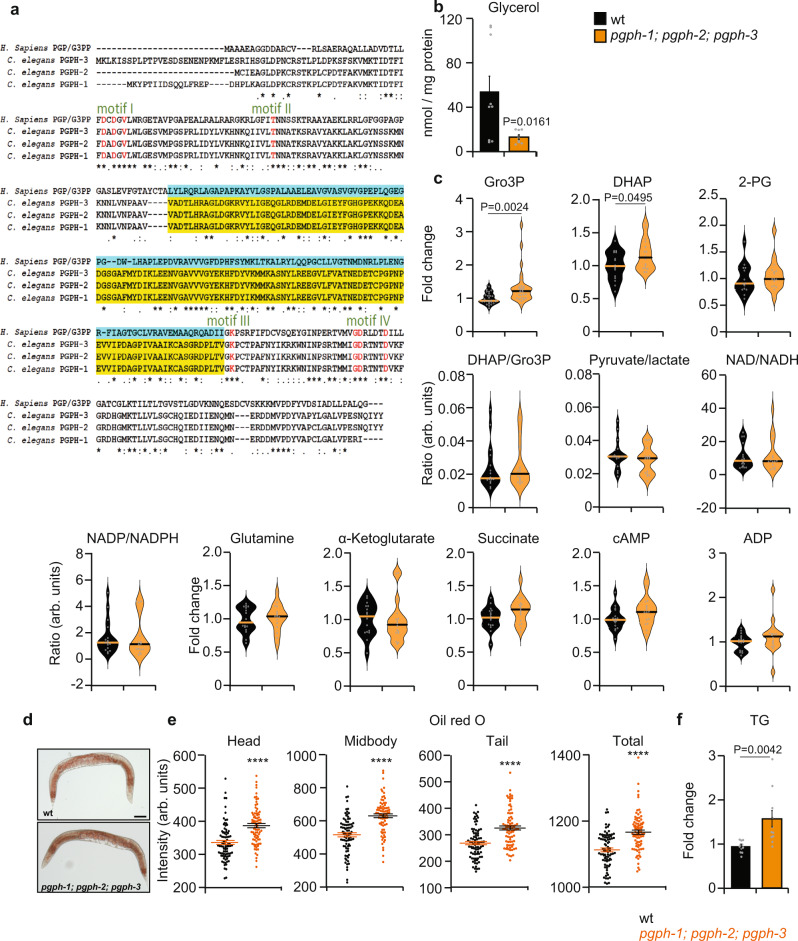
*C. elegans* harbors three homologs of mammalian PGP/G3PP with glycerol-3-phosphate phosphatase function. **a** Sequence alignment of PGP/G3PP was performed using Clustal Omega. Regulatory motifs are indicated and the cap domain is highlighted. **b** Glycerol content in wild-type (WT) and *pgph-1; pgph-2*; *pgph-3* synchronized L4/young adult animals grown on NGM plates. Data represent mean ± SEM, three biological replicates, and three independent experiments. **c** Violin plots of relative metabolite levels and ratios of some metabolites in WT and *pgph-1; pgph-2*; *pgph-3* synchronized L4/young adult animals grown on NGM plates. Lines in violin plots denote the median of the analyzed groups and gray circles indicate individual data points (four biological replicates per group per independent experiment and four independent repeats). **d**, **e** Oil red O staining and quantification in WT and *pgph-1; pgph-2*; *pgph-3* mutant 1-day adult animals. Data represent mean ± SEM from three independent experiments (WT, *n* = 83 and *pgph-1; pgph-2; pgph-3*, *n* = 81). Scale bars represent 50 µm. **f** Triglyceride levels in WT and *pgph-1; pgph-2; pgph-3* 1-day adult synchronized animals. Data represent mean ± SEM, three biological replicates per group per independent repeat, and three independent experiments. In all figures, ****P < 0.0001 and *P*-values were obtained by student’s two-tailed *t*-test. Data are provided as a Source Data file.

Using blast search, we found three homologs of G3PP in the *C. elegans* genome, *K09H11.7*, *F44E7.2*, and *C53A3.2* that we named *pgph-1*, *pgph-2*, and *pgph-3*, respectively, in accordance with the WORMBASE guidelines (Fig.1a). The three isozymes present a high level of protein sequence identity to human PGP/G3PP, with highly conserved catalytic motifs (Fig. 1a). Transcriptional reporters of *pgph-2* and *pgph-3* demonstrate ubiquitous expression of these genes with minor differences, implying possible differential tissue-specific roles (Supplementary Fig. 1a). The expression of *pgph*-*1* was not studied in the present work due to the unavailability of *pgph-1* single mutant animals and our data revealed a minor role of this enzyme in the various studied biological functions (see below).

In order to examine the roles of the PGPH isoenzymes and to avoid compensatory effects, we first generated triple (*pgph-1; pgph-2; pgph-3)* deletion mutants using CRISPR-CAS-9 technology, that we refer to as triple *pgph* mutant animals for simplicity throughout the text. Triple *pgph* mutant animals did not show phenotypic defects in pharyngeal pumping, egg laying, brood size, or swimming behaviors (Supplementary Fig. 1b–e). PGPH isozymes have G3PP activity in vivo as markedly decreased glycerol levels were found in triple *pgph* mutant animals (Fig. 1b). Accordingly, Gro3P and dihydroxyacetone-phosphate (DHAP) levels were significantly increased in these mutants (Fig. 1c). No significant changes were seen in other glycolytic metabolites as well as Krebs cycle intermediates and adenine and pyrimidine nucleotides (Fig. 1c). Also, levels of 2-phosphoglycolate (2-PG), a metabolite linked to DNA repair and a substrate with which G3PP/PGP shows high activity in vitro, were unchanged suggesting that PGPH functions mostly as a G3PP in the worm under normal in vivo conditions (Fig. 1c). PGPH/G3PP deletion is anticipated to increase lipogenesis and fat deposition. Accordingly, triple *pgph* mutant animals showed increased oil red O staining distributed throughout the body, indicating elevated fat deposition (Fig. 1d–f). Overall, these results establish that PGPH isoenzymes in the worm act as Gro3P phosphatase in vivo under normal physiological conditions.

### Enhanced lipogenesis mediates fat accumulation in triple *pgph* mutant animals

**Fig. 2:**
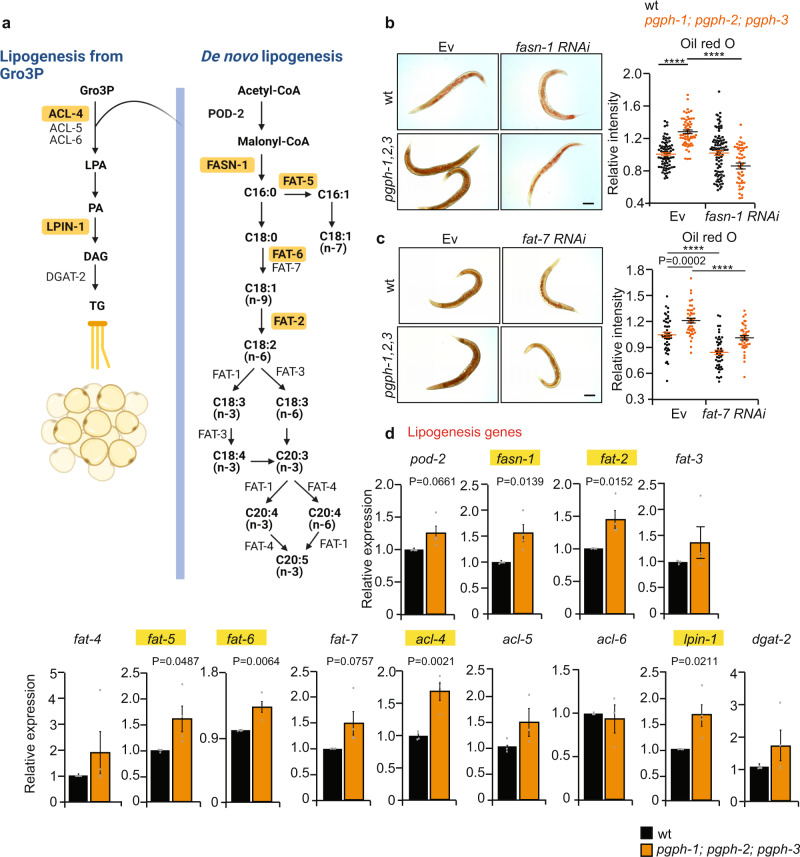
Enhanced lipogenesis is required for the fat accumulation in triple *pgph* mutant animals. **a** Scheme showing the lipogenesis pathway in *C. elegans* highlighting in yellow the genes that are significantly induced in *pgph-1; pgph-2; pgph-3* mutant animals at basal level. **b**, **c** Oil red O staining and quantification in WT and *pgph-1; pgph-2*; *pgph-*3 mutant 1-day adult animals treated or not with *fasn-1* RNAi (**b**) or *fat-7* RNAi (**c**). Scale bars indicate 50 µm. Sample numbers for **b**: *n* = 76 (WT-Ev), *n* = 59 (*pgph-1; pgph-2; pgph-3*-Ev) and *n* = 93 (WT-*fasn-1* RNAi), *n* = 50 (*pgph-1; pgph-2; pgph-3* - *fasn-1* RNAi). Sample numbers for **c**: *n* = 45 (WT-Ev), *n* = 42 (*pgph-1; pgph-2; pgph-3*-Ev), and *n* = 47 (WT-*fat-7* RNAi), *n* = 39 (*pgph-1; pgph-2; pgph-3*-*fat-7* RNAi). Data represent mean ± SEM from two independent experiments. *P*-values were obtained by one-way ANOVA with the Bonferroni test. **d** Relative expression of indicated genes in WT animals and *pgph-1; pgph-2; pgph-3* synchronized young adult animals. Genes that are significantly induced are highlighted in yellow. Data represent mean ± SEM, *n* = 4 independent experiments. *P*-values were obtained by the student’s two-tailed *t*-test. Significance in all figures: ****P < 0.0001. Data are provided as a Source Data file.

The balance between lipogenesis and lipolysis is critical for metabolic homeostasis. To determine whether lipogenesis is driving fat accumulation in triple *pgph* mutant animals, we inhibited lipogenesis by RNAi-knockdown of fat synthase-1 (*fasn-1*) and the desaturase (*fat-7*), two key genes essential for fat synthesis in *C. elegans* (Fig. 2a). Importantly, knockdown of *fasn-1* completely suppressed the increased fat accumulation phenotype observed in triple *pgph* mutant animals back to Ev levels (Fig. 2b). Also, *fat-7* RNAi significantly reduced the lipid droplets in triple *pgph* mutant animals (Fig. 2a, c). Importantly, expression of many lipogenesis genes including *fasn-1*, *fat-2*, *fat-5*, *fat-6*, *acl-4*, and *lpin-1* were significantly increased in triple *pgph* mutant animals at normal growth conditions supporting that the lipogenesis branch is activated in these mutants (Fig. 2d). We also measured the expression of several lipolysis genes to see whether lipolysis is also altered in the triple *pgph* mutant animals but did not observe a significant difference in any of the tested genes (Supplementary Fig. 2). Overall, our data support the view that suppression of PGPH enzymes increases worm fat levels via lipogenesis.

### Salt stress-induced *pgph* gene expression is required for glycerol production and resistance to hyperosmotic stress

**Fig. 3:**
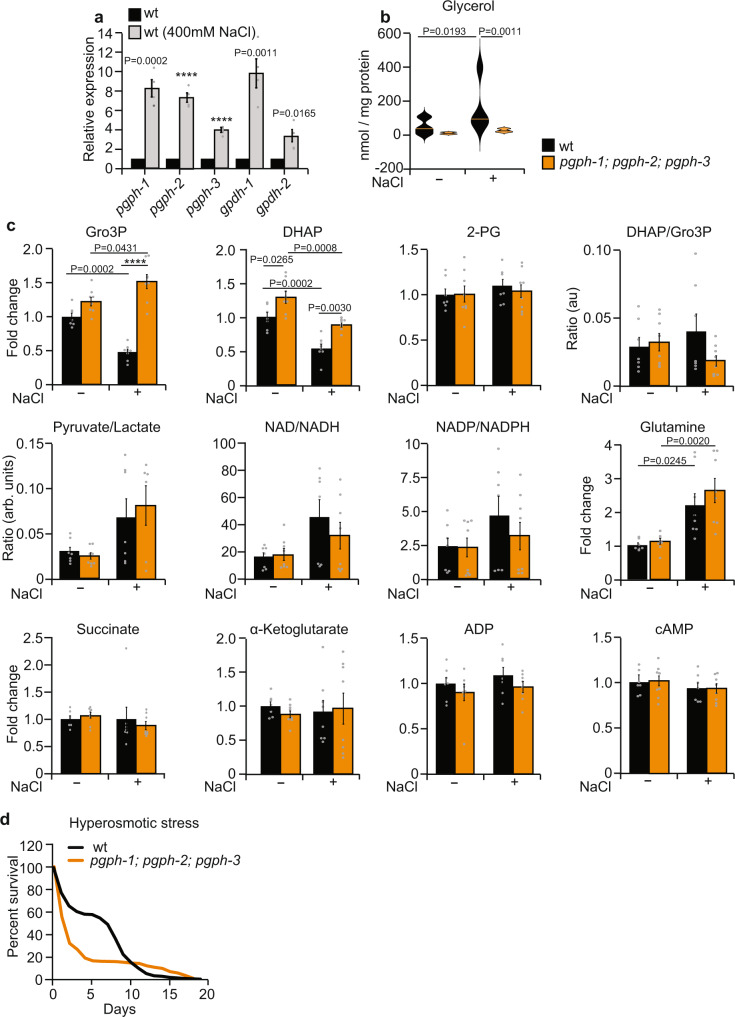
PGPH/G3PP is required for glycerol production to allow resistance to hyperosmotic stress. **a** Relative expression of indicated genes in WT animals treated or not with 400 mM NaCl for 3 h. Data represent mean ± SEM, four independent experiments. *P*-values are obtained by unpaired two-tailed student’s *t*-test. **b** Violin plots representing glycerol levels of WT and *pgph-1; pgph-2*; *pgph-3* synchronized L4/young adults exposed or not to 400 mM NaCl for 3 h. In all groups, *n* = 9. Plots are generated with three biological replicates per group per independent repeat and a total of three independent experiments. Lines in the violin plots represent median of corresponding groups. *P*-values are obtained by one-way ANOVA with the Bonferroni test. **c** Relative metabolite levels and ratios of some metabolites in WT and *pgph-1; pgph-2*; *pgph-3* synchronized L4/young adult animals grown on NGM plates and exposed or not to 400 mM NaCl for 3 h. Data represent mean ± SEM, *n* = 8, four biological replicates per group per independent repeat and two independent experiments. *P*-values are obtained by one-way ANOVA with the Bonferroni test. **d** Survival curves of indicated strains to 400 mM NaCl. *P*-values are obtained by two-sided Mantel–Cox comparisons. Number of separate experiments, animals, and detailed statistics are shown in Supplementary Data 1. Significance in all figures: ****P < 0.0001. Data are provided as a Source Data file.

We investigated whether PGPH loss abrogates glycerol production and salt stress adaptation under hyperosmotic stress, known to enhance glycerol synthesis^31,38^. Hyperosmotic (400 mM NaCl) stress markedly increased the mRNA expression of *pgph-1*, *pgph-2*, and *pgph-3* to a similar extent as *gpdh-1* (Fig. 3a). The gene expression of *pgph-1* has been previously shown to increase with high osmolarities and in *C. elegans dpy-7* mutant animals^39^. Transcriptional fluorescent reporters of *pgph-2* and *pgph-3* were also activated following NaCl stress (Supplementary Fig. 3a, b). Importantly, increased glycerol levels under hyperosmotic stress occurred only in wild-type (WT) animals and not in triple *pgph* mutants suggesting that PGPH enzymes are strictly required for glycerol synthesis under salt stress (Fig. 3b). Accordingly, Gro3P levels decreased in WT but not in the triple *pgph* mutant nematodes following salt stress, indicating a direct hydrolysis of Gro3P to glycerol in WT worms. Moreover, the increased Gro3P and DHAP levels following NaCl treatment in triple *pgph* mutant animals demonstrate that the rapid conversion of Gro3P to glycerol does not occur if PGPH enzymes are deleted (Fig. 3c). Loss of PGPH did not change 2-PG levels both at basal level and following salt stress (Fig. 3c). Hyperosmotic stress decreased lactate levels and increased the levels of some amino acids including glutamine, alanine, and aspartate. However, our metabolomics analysis revealed that salt stress-related metabolite perturbations are not associated with PGPH loss (Fig. 3c and Supplementary Fig. 3c). Because PGPH loss prevents glycerol production, an organic osmolyte critical for adaptation to hyperosmotic stress, we anticipated that the triple *pgph* mutant animals would show hypersensitivity to salt stress. Indeed, there was a pronounced decline in the survival of *pgph* mutant nematodes in the presence of NaCl (Fig. 3d and Supplementary Data 1). Overall, these data suggest that PGPH enzymes are required for glycerol synthesis and resistance to hyperosmotic stress in the worm.

### PGPH enzymes are transcriptionally regulated by *ELT-2* / GATA transcription factor

To determine how PGPH enzymes are transcriptionally regulated upon stresses that require a fast glycerol production such as hyperosmotic stress, we conducted an RNAi screen using the transcription factor RNAi library. Using GFP transcriptional reporter strains for *pgph-2* and *pgph-3*, we individually suppressed the expression of 405 transcription factors by RNAi feeding and screened for reduced GFP fluorescence (Supplementary Fig. 4). We identified 7 transcription factors capable of regulating the promoter activity of both *pgph-2* and *pgph-3* following salt treatment, *fkh-6*, *elt-2*, *ets-5*, *unc-120*, *nhr-89*, *taf-7.1*, and *daf-16* (Supplementary Fig. 4b and Supplementary Data 7). ELT-2 is required for osmotic stress resistance and the regulation of *gpdh-1* involved in Gro3P production^32^. Confocal imaging of young adult *pgph-2* and *pgph-3* transcriptional reporter (GFP expressing) strains exposed to *elt-2* RNAi revealed that RNAi-knockdown of *elt-2* strongly suppressed both *pgph-2* and *pgph-3* promoter activities (Supplementary Fig. 4c). These results identify transcriptional regulators of *pgph-2* and *pgph-3* expression following salt stress and a role of ELT-2 in *pgph* gene regulation and glycerol production.

### Glucose increases *pgph*-*1*/*pgph*-2 expression and glycerol release and enhances survival to salt stress

**Fig. 4:**
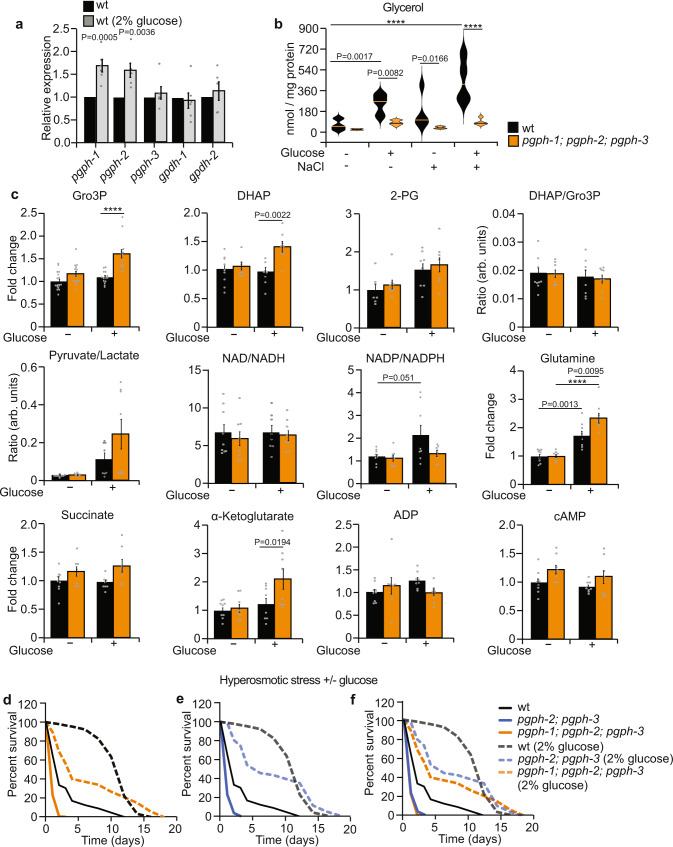
Glucose induces *pgph-1/pgph-2* gene expression, stimulates Gro3P and glycerol release in a PGPH-dependent manner, and enhances survival to salt stress. **a** Relative expression of indicated genes in synchronized L4/young adult WT animals grown on NGM plates or plates supplemented with 2% glucose. Data represent mean ± SEM, six independent experiments. *P*-values are obtained by two-tailed student’s *t*-test. **b** Violin plots representing glycerol levels of WT and *pgph-1; pgph-2*; *pgph-3* synchronized L4/young adults grown on NGM plates or plates supplemented with 2% glucose. On the day of the experiment, the two groups were either treated or not (for control) with 400 mM NaCl for 3 h. Pellets were then collected, washed with M9 buffer with adjusted osmolarities, and flash frozen in liquid nitrogen for glycerol extraction and quantification. In all groups, *n* = 9. Plots are generated with three biological replicates per group per independent repeat and a total of three independent experiments. Lines in the violin plots represent the median of corresponding groups. *P*-values are obtained by one-way ANOVA with the Bonferroni test. **c** Relative metabolite levels and ratios of WT and *pgph-1; pgph-2*; *pgph-3* synchronized L4/young adults grown on NGM plates or plates supplemented with 2% glucose. Data represent mean ± SEM, *n* = 8 biological replicates, four biological replicates per group per independent repeat, and two independent experiments. *P*-values are obtained by one-way ANOVA with the Bonferroni test. **d**-**f** Survival curves of indicated strains to 400 mM NaCl pretreated or not with 2% glucose. Number of separate experiments, animals, and detailed statistics are shown in Supplementary Data 1. *P*-values were obtained with two-sided Mantel–Cox test. In all figures: ****P < 0.0001. Data are provided as a Source Data file.

Glucose feeding increases glycerol production in *C. elegans*^14^. In order to ascertain whether PGPH enzymes are necessary for glycerol synthesis in general, or only under specific conditions such as salt stress, WT animals were exposed to 2% glucose and glycerol formation was measured. Glucose increased the transcription of *pgph-1* and *pgph-2* genes (Fig. 4a) and led to a better survival following salt stress (Fig. 4d). Importantly, loss of PGPH enzymes suppressed the glucose-induced glycerol production demonstrating that these enzymes are required for glycerol synthesis from glucose (Fig. 4b). Strikingly, prior exposure of WT animals to glucose followed by treatment with 400 mM NaCl strongly induced glycerol production, whereas the triple *pgph* mutant animals displayed only limited glycerol production under these conditions (Fig. 4b). Gro3P and DHAP levels accumulated in triple *pgph* mutant animals following glucose stress while no major changes were seen in other metabolites (Fig. 4c and Supplementary Fig. 5a). Accordingly, WT animals pretreated with 2% glucose displayed better survival under hyperosmotic stress in comparison to the non-treated WT group. Glucose excess that favors glycerol production, protected the double and triple *pgph* mutants from salt stress (Fig. 4d and Supplementary Data 1), suggesting that when G3PP activity is deleted some glycerol can still be produced upon salt stress via the classical lipogenesis-lipolysis pathway. These results may also imply that glucose may increase the survival to hyperosmotic stress by mechanisms beyond glycerol synthesis such as increased energy, production of other osmolytes such as sorbitol and other possibilities. In fact, the short-term glucose exposure may increase the osmolarity in the plates and precondition the worms to hyperosmotic stress, thereby activating protective mechanisms such as the unfolded protein response and ER hormesis. Overall the results demonstrate that: (a) glycerol formation correlates with PGPH/G3PP expression; (b) PGPH enzymes catalyze Gro3P hydrolysis under control, salt stress, and glucotoxic conditions; and (c) G3PP activity is required for resistance to hyperosmotic stress via glycerol production.

### *pgph*-2 is the primary PGPH isozyme contributing to Gro3P hydrolysis

To determine which of the three PGPH isoenzymes plays a prominent role in the worm glycerol production, we used a comparative approach between a double *pgph* mutant (*pgph-2; pgph-3*) strain generated by CRISPR-CAS9, the triple *pgph* mutant strain *(pgph-1; pgph-2; pgph-3)* and single *pgph-2* and *pgph-3* mutant worms. The specific role of *pgph-1* was not directly investigated with a single mutant strain because the high sequence identity between *pgph-1* and the other two isoforms prohibited the generation of a specific CRISPR deletion mutant. However, the genetic comparison of the survival to hyperosmotic stress, glycerol levels, and Gro3P measurements in double and triple *pgph* mutant animals implied a less important role for *pgph*-*1* for the following reasons. First, we did not observe any defect in physiological parameters including pharyngeal pumping, egg laying, as well as brood size in the double *pgph* mutant strain (Supplementary Fig. 6a–c), similar to what we have noticed with the triple *pgph* mutant strain (Supplementary Fig. 1a–c). Under normal growth conditions, glycerol levels were only significantly reduced in the triple *pgph* mutant animals with a reduction trend in double *pgph* mutant (Supplementary Fig. 6d). As previously demonstrated (Fig. 3b and Fig. 4b), WT animals exhibited a dramatic increase in glycerol levels following salt stress, glucose stress, or the two stresses simultaneously (Supplementary Fig. 6e, f). However, the extent of reduction in glycerol levels was similar between double and triple *pgph* mutant animals under these conditions, indicating that lack of *pgph-1* does not cause a stronger defect in glycerol synthesis (Supplementary Fig. 6e, f). Accordingly, Gro3P levels in double and triple *pgph* mutant animals were not significantly different under normal growth conditions or following hyperosmotic stress or excess glucose (Supplementary Fig. 6g–i). Oil red O staining revealed a similar increase in fat levels in the double and triple *pgph* mutants, driven by elevated lipogenesis (Supplementary Fig. 6j). Moreover, double and triple *pgph* mutant animals were equally hypersensitive to hyperosmotic stress (Supplementary Fig. 6k-m). Pre-treatment of the worms with 2% glucose or 1% glycerol increased the survival of the animals to 400 mM NaCl (Fig. 4d–f and Supplementary Fig. 6n–p and Supplementary Data 1) and both double and triple *pgph* mutant animals pretreated with 2% glucose or 1% glycerol were hypersensitive in comparison to the treated WT group (Fig. 4d–f and Supplementary Fig. 6n–p and Supplementary Data 1). However, triple *pgph* mutant animals lacking *pgph-1* in comparison to the double mutant animals were slightly more hypersensitive to salt stress, suggesting that *pgph*-*1* may play a small role under conditions with excess Gro3P precursors (Fig. 4f and Supplementary Fig. 6p). Overall, the data indicate that *pgph*-*1* likely plays a minor role in glycerol synthesis, fat accumulation, and survival to hyperosmotic stress.

Using single mutant strains, loss of *pgph-2* alone in two different alleles mimicked the salt stress hypersensitivity observed in the double and triple *pgph* mutant animals (Supplementary Fig. 6k and Supplementary Data 1). In contrast, *pgph-3(tm3391)* mutant animals were not sensitive to salt stress (Supplementary Fig. 6l and Supplementary Data 1). Moreover, in 1-day adult animals, *pgph-2* was found to be transcriptionally activated in the intestine following NaCl stress, while *pgph-3* activation occurred mostly in embryos (Supplementary Fig. 3b). Overall, our data point towards a more prominent role for *pgph-2* and a less important role for *pgph*-*1* and *pgph-3*. Our results demonstrate that PGPH isoenzymes in the worm, in particular *pgph*-*2*, act as Gro3P phosphatase for glycerol synthesis under both normal conditions and under salt stress and are required for survival to hyperosmotic stress.

### PGPH loss shortens lifespan and healthspan in normal and high glucose conditions

**Fig. 5:**
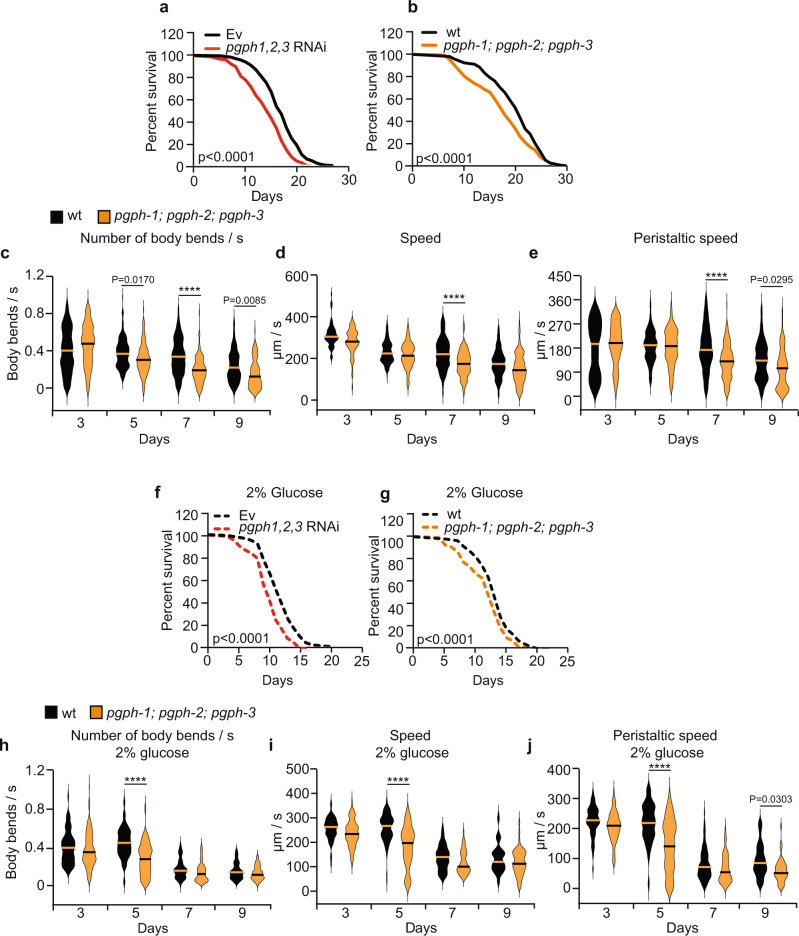
PGPH loss shortens lifespan and healthspan in normal and high glucose conditions. **a** Lifespan survival curves of WT animals treated with Ev or *pgph* RNAi. **b** Lifespan survival curves of indicated worm strains on NGM plates. **c**–**e** Locomotion analysis of worm body bends/s (**c**), speed (**d**), and peristaltic speed (**e**) of indicated worm strains at days 3, 5, 7, and nine of age. Data is represented using violin box plots from three independent experiments with indicated median lines. More than 150 tracks are analyzed per condition and an exact number of tracks is shown in the data source. **f**, **g** Survival curves of indicated worm strains grown on plates supplemented with 2% glucose. **h**–**j** Locomotion analysis of worm body bends/s (**h**), speed (**i**), and peristaltic speed (**j**) of indicated worm strains grown on plates supplemented with 2% glucose at days 3, 5, 7, and 9 of age. Data is represented using violin box plots from three independent experiments with indicated median lines. More than 150 tracks were analyzed per condition and an exact number of tracks is shown in the data source. Number of separate lifespan and glucotoxicity experiments, animals, and detailed statistics are shown in Supplementary Data 2 and 3. Significance in all figures: ****P < 0.0001. *P*-values of lifespan and glucotoxicity experiments were obtained by two-sided Mantel–Cox. *P*-values of locomotion analysis were obtained by one-way ANOVA with the Bonferroni test. Data are provided as a Source Data file.

Excess glucose is known to induce glucotoxicity as well as early onset of aging-related abnormalities^14^. Loss of PGPH enzymes, via either RNAi or double and triple deletion mutations, significantly reduced the median lifespan of *C. elegans* (about 12%, see Supplementary Data 2), supporting the view that it plays a protective role in aging and healthy aging (Fig. 5a, b and Supplementary Fig. 7d and Supplementary Data 2). We next investigated the influence of PGPH loss on movement behaviors, an index of healthy aging, in WT and *pgph* mutants. Locomotion depends on the integrity of the worm neuromuscular function^40–42^, and a decline in movement reflects a deterioration in health, normally observed after day 5 in WT animals^43–47^. Motility of the aging *pgph* mutant animals in terms of number of body bends per second, speed, and the peristaltic speed, deteriorated faster as compared to WT nematodes (Fig. 5c–e and Supplementary Fig. 7e, f). Recent reports indicate that increased stress resistance and improved locomotion in aging *C. elegans* animals are indicative of extended health span and healthy aging^46^. We measured the ability of the double and triple *pgph* mutant animals to resist various stresses. Reduced PGPH enzymes expression via RNAi increased the susceptibility to oxidative stress, and delayed recovery after 24 h hypoxia and 16 h cold stress (Supplementary Fig. 7a–c and Supplementary Data 4-6). Obesity linked to excess carbohydrate reduces lifespan and healthspan in many organisms^14,48–52^. We next determined whether loss of PGPH exacerbates the lifespan decline caused by excess glucose. Loss of all three PGPH enzymes significantly reduced median lifespan (about 7–16%; see Supplementary Data 3) upon glucose treatment, supporting a protective role for PGPH enzymes in the presence of excess glucose (Fig. 5f, g and Supplementary Fig. 7g). Accordingly, movements deteriorated faster during aging in *pgph* mutant animals in comparison to WT upon glucose feeding (Fig. 5h–j and Supplementary Fig. 7h, i). These results support a role for PGPH enzymes in aging, particularly healthy aging, as well as in combating various stresses.

### *pgph*-2 overexpression improves healthspan and decreases fat content

**Fig. 6:**
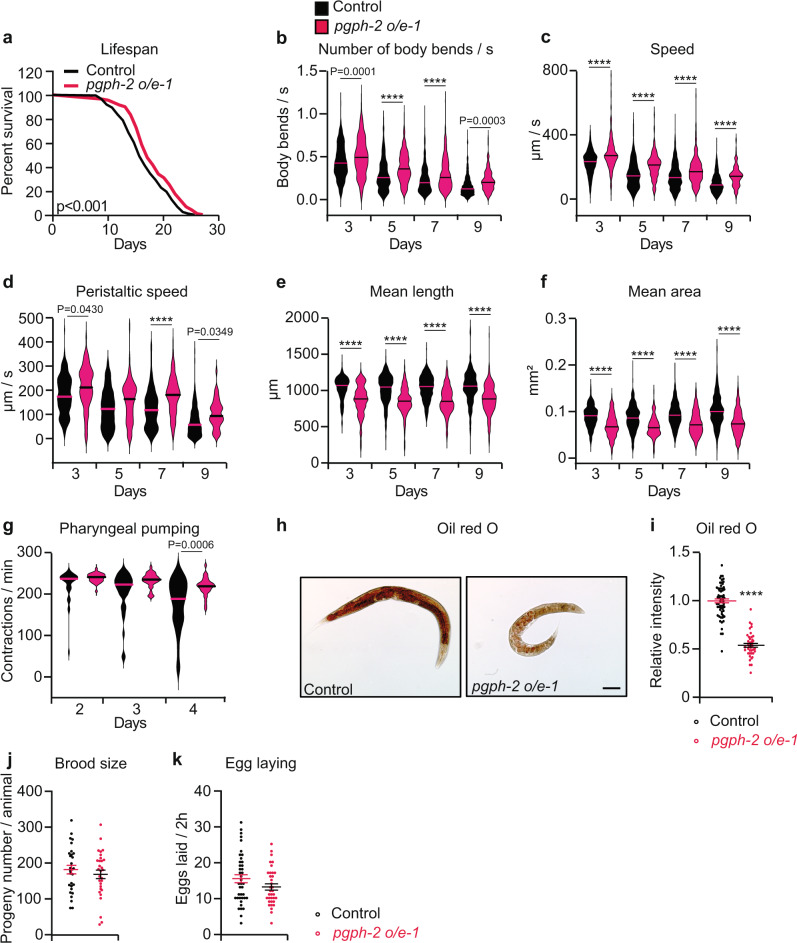
*pgph*-2 overexpression improves healthspan and decreases fat content. **a** Lifespan of *pgph-2* overexpressing animals in comparison to control nematodes. Number of separate lifespan experiments, animals and detailed statistics are shown in Supplementary Data 2. *P*-value is obtained using the two-sided Mantel–Cox test. **b**–**f** Locomotion and body size analyses including the number of body bends per second (**b**), speed (**c**), peristaltic speed (**d**), mean length (**e**), and mean area (**f**) in *pgph-2* overexpressing animals in comparison to control nematodes at days 3, 5, 7, and 9 of age. Data is shown using violin box plots with denoted median lines from three independent experiments. More than 150 tracks analyzed per condition and exact number of tracks is shown in data source. *P*-values are obtained by one-way ANOVA with Bonferroni test. **g** Violin plots showing pharyngeal pumping rates of control and *pgph-2* overexpressing nematodes at days 2, 3, and 4 of age. Sample numbers: *n* = 35 (control day 2), *n* = 27 (*pgph-2 o/e1* day 2), *n* = 37 (control day 3), *n* = 30 (*pgph-2 o/e1* day 3), *n* = 36 (control day 4), *n* = 30 (*pgph-2 o/e1* day 4). *P*-values are obtained by one-way ANOVA with Bonferroni test. **h**, **i** Oil red O staining (**h**) and quantification (**i**) in 3 days old adult control and *pgph-2* overexpressing nematodes. Scale bars represent 50 µm. Data represent mean ± SEM from three independent experiments. Sample numbers: *n* = 66 (control), *n* = 44 (*pgph-2 o/e1*). *P*-value obtained with two-tailed student’s *t*-test. **j**, **k** Brood size (**j**) and egg laying (**k**) in *pgph-2* overexpressing animals in comparison to control nematodes. *P*-value obtained with two-tailed student’s *t*-test. Data represent mean ± SEM *n* = 10, three independent experiments. Significance in all figures: ****P < 0.0001. Data are provided as a Source Data file.

There is a growing interest in interventions that compress the years of decay and disease and extend the years of healthy living, rather than extending maximal lifespan^25,52–54^. Considering that *pgph* mutant worms showed accelerated aging and declined healthspan in normal as well as excess glucose conditions, along with susceptibility to various stresses, we investigated whether the overexpression of PGPH enzymes protects against the above parameters. We generated transgenic worms overexpressing either *pgph-2* or *pgph-3* and assessed lifespan and healthspan parameters. Overexpression of *pgph-2* led to a small but significant increase in median lifespan in three separate transgenic lines (about 6%; Supplementary Data 2) under control non-stress condition (Fig. 6a and Supplementary Fig. 8a, b). The three transgenic lines showed mild overexpression of no more than three fold (Supplementary Fig. 7j). Video imaging of transgenic animals overexpressing *pgph-2* revealed that they maintain a younger age-associated locomotion behavior for a longer time in comparison to Control animals *WT* animals (Supplementary movies 1–4 taken at day 9). The number of body bends per second, speed, and peristaltic speed were significantly higher in the *pgph-2* overexpressing animals with increasing age, supporting a role for this enzyme in the prevention of age-related decline in motor function (Fig. 6b–d and Supplementary Fig. 8c–f).

Dietary restriction in *C. elegans* is known to reduce body size and decrease fat content^55^. Because PGPH hydrolyzes Gro3P, which is the backbone for the synthesis of glycerolipids, we anticipated that *pgph-2* overexpressing worms would display decreased lipogenesis and accumulate less fat with age. Indeed, *pgph-2* overexpression reduced body length and mean body surface area (Fig. 6e, f and Supplementary Fig. 8g, h) and decreased fat content in three separate transgenic lines (Fig. 6h, i and Supplementary Fig. 8k). *C. elegans* feeding depends on the rhythmic contraction of the pharynx known as pharyngeal pumping, a primary indicator of food intake. Pharyngeal pumping rates in *pgph-2* overexpressing animals were unchanged demonstrating that the decreased fat content is not due to a decrease in calorie intake (Fig. 6g and Supplementary Fig. 8i, j). Moreover, egg laying and brood size were not altered in *pgph-2* overexpressing lines suggesting that the extension in healthspan is not at the expense of energy spent on reproduction (Fig. 6j, k and Supplementary Fig. 8l, m). Similar experiments carried out with animals overexpressing *pgph-3* did not show differences in any of the above-measured phenotypes. Overall, these results indicate that *pgph-2* overexpression, but not *pgph-3*, slightly extends medium lifespan, but has significant beneficiary effects in delaying age-related decline in locomotor function and in preventing fat accumulation.

### *pgph*-2 overexpression increases lifespan in the presence of glucose and reduces glucose-induced fat deposition via reduced lipogenesis

**Fig. 7:**
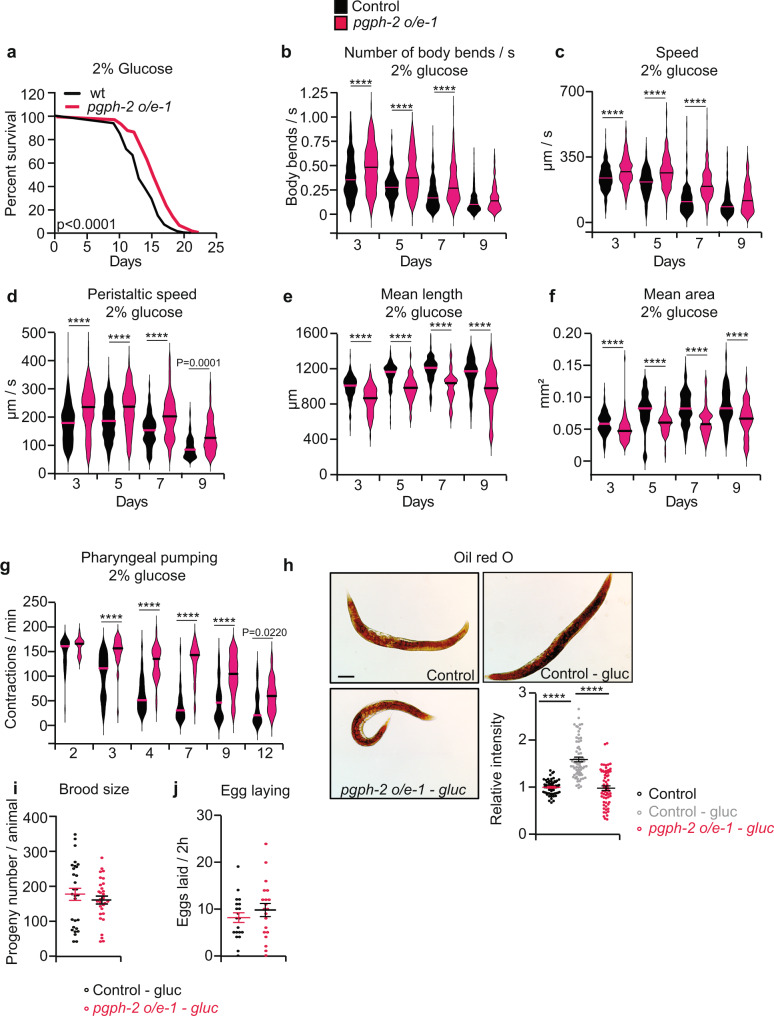
*pgph*-2 overexpression increases lifespan under excess glucose and reduces glucose-induced fat deposition. **a** Survival curves of *pgph-2* overexpressing animals in comparison to control nematodes in excess glucose conditions. Number of separate experiments, animals, and detailed statistics are shown in Supplementary Data 3. *P*-values are obtained by the two-sided Mantel–Cox test. **b**–**f** Locomotion and body size analyses including the number of body bends per second (**b**), speed (**c**), peristaltic speed (**d**), mean length (**e**), and mean area (**f**) in *pgph-2* overexpressing animals in comparison to control nematodes grown on plates supplemented with 2% glucose at days 3, 5, 7, and 9 of age. Data is represented using violin box plots with denoted median lines from three independent experiments. More than 150 tracks were analyzed per condition and an exact number of tracks is shown in the data source. **g** Violin box plots showing pharyngeal pumping rates of control and *pgph-2* overexpressing nematodes grown on plates supplemented with 2% glucose at the indicated age. Sample numbers: *n* = 36 (control day 2), *n* = 30 (*pgph-2 o/e1* day 2), *n* = 34 (control days 3, 4, 7, 9, 12), *n* = 30 (*pgph-2 o/e1* days 3, 4, 7, 9, 12). **h** Oil red O staining and quantification in 3 days old adult control and *pgph-2* overexpressing nematodes grown on plates supplemented with 2% glucose. Scale bars represent 50 µm. Data represent mean ± SEM from three independent experiments. Sample numbers: *n* = 65 (control), *n* = 68 (control-2% glucose), *n* = 63 (*pgph-2 o/e1*). **i**, **j** Brood size (**i**) and egg laying (**j**) in indicated *pgph-2* overexpressing animals grown on plates supplemented with 2% glucose in comparison to control nematodes. Data represent mean ± SEM, *n* = 10, three independent experiments. Significance in all figures: ****P < 0.0001. From **b**–**j**, *P*-values are obtained by one-way ANOVA with the Bonferroni test. Data are provided as a Source Data file.

Three separate transgenic lines overexpressing *pgph-2* showed a significant increase (about 20%) in medium lifespan in the presence of glucose (Fig. 7a and Supplementary Fig. 9a, b, Supplementary Data 3). Video imaging of glucose-fed *pgph-2* overexpressing animals revealed that they maintained a better locomotion behavior with age (Supplementary movies 5–8 at day 9). Specifically, the number of body bends per second, speed, and peristaltic speed were much improved in the glucose-fed *pgph-2* overexpressing animals (Fig. 7b–d and Supplementary Fig. 9c–f). Furthermore, *pgph-2* overexpression reduced body length and mean body surface area in glucose excess conditions (Fig. 7e, f and Supplementary Fig. 9g, h). Glucose supplementation increased fat content in control animals, whereas animals with *pgph-2* overexpression displayed lower fat levels that were comparable to control animals grown on normal growth medium (Fig. 7h and Supplementary Fig. 9k). Importantly, the expression of several lipogenesis genes was significantly decreased in *pgph-2* overexpressing animals in comparison to the controls, supporting that lipogenesis is negatively regulated in *pgph-2* overexpressing animals, specifically under excess glucose conditions (Supplementary Fig. 11a). Also, we did not observe a significant increase in any of the tested lipolysis genes, suggesting that the decrease in fat deposition in *pgph-2* overexpressing animals is unlikely driven by an increased lipolysis, especially following excess glucose (Supplementary Fig. 11b).

Animals overexpressing *pgph-2* maintained higher pharyngeal pumping rates than control animals, with increasing age, indicating that the decrease in fat deposition is not due to reduced food intake, and that the overexpression of *pgph-2* delays age-related decline in food intake (pharyngeal pumping) under glucotoxic conditions (Fig. 7g and Supplementary Fig. 9j). Additionally, brood size and egg laying were not altered in *pgph-2* overexpressing lines grown in excess glucose conditions indicating that the increased lifespan and healthspan in the glucose-rich medium are not due to a decrease in reproduction (Fig. 7i, j and Supplementary Fig. 9l, m). In contrast, overexpression of *pgph-3* alone did alter lifespan and did not improve healthspan parameters in glucose excess conditions (Supplementary Fig. 10).

Interestingly, these beneficial effects of *pgph*-2 overexpression tend to be more prominent under glucotoxic stress than normal growth conditions (Supplementary Data 9). In fact, a comparison of survival, locomotion parameters, as well as pharyngeal pumping in three transgenic lines overexpressing *pgph-2* reveal larger beneficial effects in the presence of glucose in comparison to the absence of glucose (Supplementary Fig. 12). What may be viewed as a potential caveat in these series of experiments on healthy aging parameters in the absence and presence of glucotoxicity should be mentioned. Thus, in *pgph-2*-overexpression studies, while many of the phenotypes on days 3 and 7 are more prominent in the presence of 2% glucose, some effects occur for animals without the addition of glucose for many phenotypes at days 3 and 9. How can this be explained? One possibility is that a relative small increase in *pgph-2* overexpression (2.5 fold at mRNA level) is enough to have beneficial effects until day 7 in the presence 2% glucose but to a lesser extent at day 9 when glucotoxicity is more chronic and therefore more deleterious. Indeed, control animals exposed to 2% glucose have a medium and maximum lifespan of 13 and 20 days, respectively, compared to 16 and 27 days without glucose. Perhaps higher *pgph-2* overexpression could lead to higher beneficial effects also at day 9 but this remains to be tested. Overall, enhanced PGPH-2  activity, unlike PGPH-3, protects from age-related decline and fat accumulation in excess glucose conditions.

## Discussion

**Fig. 8:**
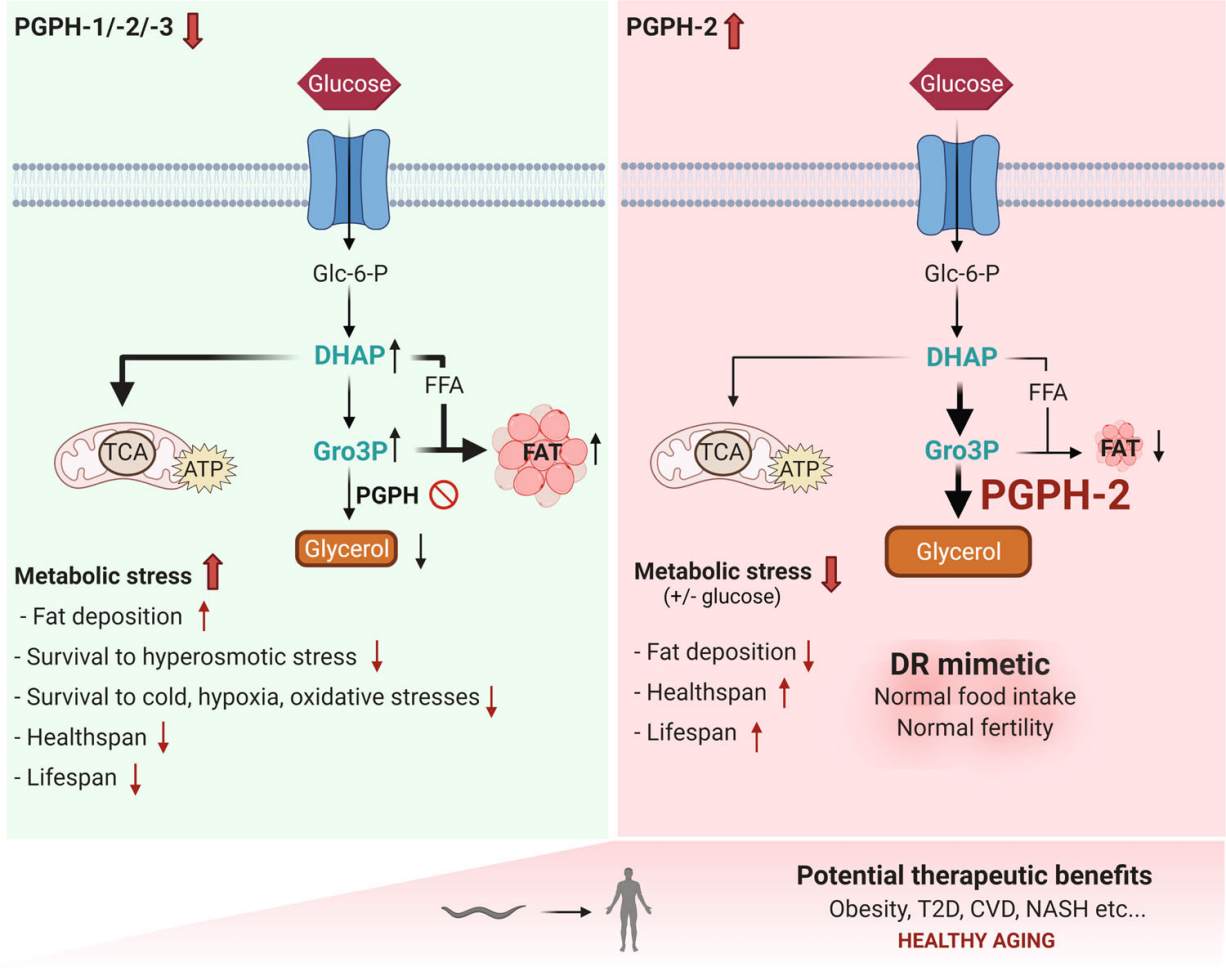
Graphical abstract summarizing the main findings. We have characterized the function of the PGPH enzymes in *C. elegans*. PGPH enzymes act as glycerol-3-phosphate phosphatase (G3PP) and are responsible for glycerol production via the direct hydrolysis of glycerol-3-phosphate. Loss of PGPH enzymes decreases resistance to diverse stresses, shortens lifespan and healthspan and exacerbates glucotoxicity. In contrast, the overexpression of PGPH-2, mimics calorie restriction without changes in food intake and fertility, decreases fat deposition, protects from glucose-induced toxicity and promotes healthy aging. Overall, the results identify G3PP as a candidate therapeutic avenue to treat obesity and age-related cardiometabolic disorders, and to promote healthy aging.

Here, we used *C. elegans* as a model to identify the biological functions of G3PP. We demonstrate that *C. elegans* harbors three PGPH isozymes with G3PP activity in vivo. We also show that PGPH enzymes regulate glycerol and fat metabolism, organismal adaptation to various stresses, in particular hyperosmotic stress and glucotoxicity, as well as healthy aging (Graphical abstract and Fig. 8).

Multiple lines of evidence demonstrate that PGPH acts as a G3PP in vivo in *C. elegans*. Thus, metabolomic measurements show that both Gro3P and DHAP are increased under normal conditions upon PGPH enzymes deletion and more prominently following salt and glucose stresses, while other metabolites, including 2-PG, are not perturbed. Moreover, *pgph* double and triple mutant animals show reduced levels of glycerol under basal conditions and are incapable of inducing glycerol production following salt, glucose, or both stresses together. Importantly, we also show that fat levels are increased in triple *pgph* mutant animals under basal conditions, in accordance with a rise in Gro3P levels.

The role of glycerol in organismal adaptation to hyperosmotic stress is well established. In *C. elegans*, GPDH which converts DHAP to Gro3P, is induced in response to acute salt stress and is required for glycerol production for hyperosmotic stress adaptation^31–35,38^. However, the enzyme responsible for the hydrolysis of Gro3P to produce glycerol has not been identified. High osmolarities have been recently shown to induce *pgph-1* gene expression^39^. Here, we demonstrate that *pgph* genes are strongly induced following hyperosmotic and glucotoxic stresses and are required for glycerol production and survival under these stresses.

Besides Gro3P and 2-PG, PGP/G3PP has also been shown to detoxify certain toxic glycolytic side products in cancer cell lines, particularly 4-phosphoerythronate and 2-phospholactate, produced by glyceraldehyde 3-phosphate dehydrogenase and pyruvate kinase, respectively^8,56^. Upon their accumulation following *pgp* deletion, glycolysis, the pentose phosphate pathway, and ATP production are altered^8,57^. However, our work reveals no change in the levels of key glycolytic and Krebs cycle intermediates, adenine and pyrimidine nucleotides, linked to energy metabolism, except Gro3P and DHAP, with PGPH loss at basal level and after salt or glucose treatment. In addition, *pgph* mutant worms are viable and under normal growth conditions do not show any physiological defects, indicating that the accumulation of toxic side-metabolites does not occur to any significant effect in the *pgph* mutant worm. Other enzymes than PGPH may remove toxic metabolite side products in *C. elegans*.

We also addressed the role of G3PP in the protection from glucotoxicity and its function related to healthy aging (Figs. 5–7). We show that excess glucose, known to shorten lifespan in *C. elegans*^14,16,48,58^, induces PGPH enzymes expression and stimulates glycerol release. Importantly, loss of PGPH exacerbates glucotoxicity, slightly decreases median lifespan and significantly reduces healthspan, as evidenced by a decline in locomotion parameters and the increased susceptibility of worms to various stresses, such as hypoxia, cold and oxidative stresses.

Glucotoxicity in *C. elegans* has been linked to various metabolic derangements including AMPK inactivation^16^, inhibition of DAF-16 and HSF-1 and downregulation of aquaporin gene expression^14^, glycogen, and fat accumulation^48,49^. We propose that the exacerbation of glucotoxicity by loss of PGPH is due to the accumulation of Gro3P and associated enhanced lipogenesis, excess phospho-glycerolipids, and TG deposition, as well as ROS production through enhanced Gro3P shuttle^59^. In line with these possibilities, decreased Gro3P levels have been reported in long-lived mutants^60^.

On the other hand, interventions that prevent damage caused by hyperglycemia and obesity-related disorders, extend lifespan and healthspan in various organisms^15–26^. We now show that the overexpression of *pgph-2* protects from glucose-induced toxicity and improves healthspan possibly by preventing lipogenesis and ROS production by the Gro3P shuttle. Fat accumulation in non-adipose tissues causes mitochondrial dysfunction and age-related metabolic decline^61^. Specifically, fat accumulation in ectopic tissues shortens lifespan whereas lipid hydrolysis in *C. elegans* extends lifespan^62–66^. Furthermore, interventions that promote longevity, such as reduced insulin signaling and dietary restriction, are associated with a reduction of ectopic fat in the worm^67^. Our data suggest that by enhancing the conversion of Gro3P to glycerol, *pgph-2* overexpression decreases lipogenesis, Gro3P shuttle activity, and ROS production resulting in an extension of healthspan and median lifespan under glucose-rich conditions. Precisely, we show that lipogenesis is increased by the suppression of the PGPH enzymes and reduced by overexpressing *pgph*-2. Additional work is needed to precisely identify the mechanism(s) whereby variations in G3PP levels modulate healthspan.

One limitation of this study is the minimal information obtained to elaborate on the individual role of *pgph*-*1* *in C. elegans* biological processes. The high identity in the genetic sequences between the three PGPH enzymes and the close proximity on chromosome V made it not possible to obtain single *pgph-1* mutant animals. Nonetheless, our comparative approach between the double and triple *pgph* mutant animals as well as *pgph-2* and *pgph-3* single deletion mutants indicates that *pgph*-2 plays a major role in glycerol production, hyperosmotic stress response, aging, and glucotoxicity, presumably because of its prominent transcriptional regulation in the intestine, unlike PGPH-3. Moreover, although *pgph-1* expression levels are induced by salt and glucose stresses, the fact that the glycerol content, the Gro3P levels, and hyperosmotic stress resistance phenotypes are not different between *pgph* double *(pgph-2; pgph-3)* and triple *(pgph-1; pgph-2; pgph-3*) mutant nematodes indicates that *pgph*-*1* is not as important as *pgph*-*2* for Gro3P phosphatase activity and resistance to hyperosmotic stress.

Calorie restriction is one of the most robust health-promoting interventions yet difficult to sustain^15–26^. Researchers have looked for pharmacological calorie restriction mimetics to promote calorie restriction benefits without the discomfort linked to strict diets to treat cardiometabolic disorders^68^. Interestingly, this work sheds light on a distinct pathway that mimics in part the beneficial effects of calorie restriction without the restriction of calorie intake and changes in fertility. Specifically, this work highlights a significant resemblance in terms of better healthspan parameters and reduced fat accumulation and size, between *pgph-2* overexpression and effects of calorie restriction regimens. However, although *pgph*-2 overexpression significantly increases lifespan under normal conditions and glucotoxic stress, the extension of lifespan is much more prominent with calorie restriction or in insulin-like signaling pathway mutants in *C. elegans*^20,69^.

In conclusion, the results show marked effects of variations in PGPH/G3PP levels in terms of healthspan but modest effects on lifespan *in C. elegans*. However, it should be pointed out that improved healthspan does not need to correlate with large increases in lifespan, and the large lifespan increase in some *C. elegans* mutants does not scale up to mammals or primates at all, but the healthspan does well. Hence, it may be hypothesized that G3PP activation might provide an entirely novel approach to prevent and treat diseases due to nutritional excess such as obesity, diabetes, NASH, and CVD. In fact, a recent study has identified G3PP as a new potential candidate longevity gene via an exome-wide association study in a long-lived centenarian cohort in comparison to younger individuals^70^. Thus, in the worm, enhanced G3PP activity protects from fat deposition and promotes healthy aging, and aging in normal and excess glucose conditions, which is by far the most single contributor to cardiometabolic disorders.

## Methods

### *C. elegans* strains, maintenance, and RNAi interference

Nematodes were maintained and synchronized using standard culture methods^71^. We used the following strains: N2 Bristol, *pgph-2p::GFP* (rCesF44E7.2::GFP + pCeh361), *pgph-3p::GFP (rCesC53A3.2::GFP* + *pCeh361)* that were obtained from the *Caenorhabditis* Genetics Center. *pgph-3(tm3391)* was provided by Dr. Mitani (Nation bio-resource project of the Mext, Japan). *pgph-2(xq830-1), pgph-2(xq830-2), pgph-2(xq830); pgph-3(tm3391)* and *pgph-1(xq829); pgph-2(xq830); pgph-3(tm3391)* strains were generated in our lab using CRISPR-CAS9 technology and will be available upon request. Two RNAi clones were obtained from the Dharmacon library and RNAi feeding experiments were performed as described in^72^, and bacteria transformed with empty vector were used as control. For all RNAi experiments, phenotypes were scored with the F1 generation.

### Synchronization methods

Two synchronization methods were used in this study. For experiments that require a small number of progeny including lifespan, locomotion, glucotoxicity, stress resistance, brood size, pharyngeal pumping, and swimming behavior experiments, animals were synchronized by transferring 5–8 gravid hermaphrodites to fresh agar plates to permit egg laying for four hours, then the hermaphrodites were removed and eggs were allowed to hatch and grow until they reached the experimental stage. For biochemical experiments including RNA expression, glycerol, and metabolite measurement experiments, the nematodes were synchronized by the standard hypochlorite bleaching method^73^.

### Plasmid construction and overexpressing transgenic strains

The overexpressing *pgph-2* and *pgph-3* plasmids were generated using Clontech In-Fusion PCR Cloning Kit according to manufacturer’s protocol^74^. The promoter region and genes were amplified from N2 worm genomic DNA and were cloned into ppD49.26 vector using *sbf-1* and *kpn-1* cloning sites for *pgph-2* and *sbf-1* and *EcoR-V* for *pgph-3*. Cloning mixtures were transformed using stellar competent cells according to the manufacturer’s protocol. 10 ng/ul of plasmid DNA, 20 ng/ul mCherry, and 170 ng/ul pBluescript were injected into worm gonad arms of WT worms and the positive lines were maintained after transmission to the F3 generation.

### Transcription factor RNAi screen

Briefly, 3–5 adult worms were grown on RNAi plates and allowed to lay eggs. After 3 days, the plates were scored for reduced GFP fluorescence signal at basal conditions using a fluorescence dissecting microscope. Of their progeny, around 15 animals were handpicked and placed on corresponding RNAi plates supplemented with 400 mM NaCl for two hours. The plates were screened for reduced GFP signal and a secondary screen to confirm positive hits followed.

### Lifespan and glucose toxicity assays

All lifespan and glucose toxicity curves were performed at 20 °C. Briefly, *C. elegans* nematodes were synchronized as described above. For glucose toxicity, the animals were constantly transferred to *E. coli* seeded agar plates supplemented with 2% glucose. Three days after egg preparation, around 120 animals were manually transferred (40 animals per plate in triplicates) to fresh plates. Worms were transferred daily for the first week and every other day afterwards until the aged population is well separated from growing larvae. Worms were scored daily and were considered alive if they responded to gentle tapping with a platinum wire. Worms that crawled off the plate or died from bagging or internal hatching were censored from the analysis.

### Locomotion assays

Along with the lifespan and glucose toxicity assays, the locomotion behaviors of the nematodes were filmed for 30 s at days 3, 5, 7, and 9 using the Wormtracker system (MBF Bioscience). More than 200 tracks per genotype/condition were generated in three independent repeats. Movies were analyzed and average speed, peristaltic speed, number of body bends per second were computed using software Wormlab.

### Paraquat resistance assay

Resistance to 100 mM paraquat was performed as previously described^75^. Briefly, 4–8 synchronized L4-young adult animals were transferred to the wells of a 96 well plates containing 100 mM paraquat dissolved in M9 solution. We monitored at least 12 wells per condition.

### Hyperosmotic stress resistance assay

Hyperosmotic stress resistance was assayed as previously described^33^. Briefly, synchronized 1-day adult worms were transferred to 400 mM NaCl plates. Worms that responded by the movement to touch with the platinum wire were considered alive. Survival was measured daily.

### Hypoxia stress

One-day-old adult animals were transferred to corresponding RNAi plates and were kept in a Bio-Bag Environmental Chamber Type A (Becton Dickinson Microbiology Systems) for 24 h at 20 °C. The number of crawling animals was scored at indicated time points and recovery rates were calculated.

### Cold stress

For cold stress, 1-day adult animals were transferred to corresponding RNAi plates and were kept at 4 °C for 16 h. The plates were transferred back to 20 °C and the number of crawling animals was scored at indicated time points to calculate recovery rates.

### RNA extraction and RT-PCR

Synchronized young adult nematodes were harvested and total RNA was extracted with Trizol. Reverse transcription and qRT-PCRs were performed^3^. Transcripts were normalized to *cdc42*. Primers sequences are available in Supplementary Data 8.

### Oil red O staining

Oil red O staining was performed as previously described with small modifications^76^. Briefly, synchronized young adult animals were collected with M9 buffer and washed twice with PBS1x-0.01% triton X. Animal pellets were treated for 15 min with PBS1x, 0.01% Triton X, 60% isopropanol solution and transferred to Eppendorf tubes and incubated in 60% oil red O solution overnight. The oil red O stock solution was equilibrated for >3 days and the 60% diluted solution was equilibrated overnight and filtered two times on the day of the experiment. Images were taken using a Leica microscope and were analyzed using Image J.

### Metabolite extraction and glycerol determination

NaCl treatment: Synchronized L4/young adult animals exposed or not to 400 mM NaCl for 2 h and were harvested and washed with M9 buffer three times adjusted to match plate salinity. Pellets were flash frozen in liquid N2.

Glucose treatment: Synchronized L1 animals by sodium hypochlorite were plated on NGM plates or plates supplemented with 2% glucose for 48 h then harvested with M9 buffer and washed three times before flash freezing in liquid N2.

Glucose-NaCl treatment: Synchronized L1 animals by sodium hypochlorite were plated on NGM plates or plates supplemented with 2% glucose and grown for 48 h at 20 °C. The animals were then collected with M9 buffer with adjusted salinities and exposed to plates supplemented with 400 mM NaCl for 3 h. Animals were harvested with M9 buffer and washed three times before flash freezing in liquid N2.

Glycerol levels were determined using a radiometric glycerol assay using [γ-^32^P]ATP and glycerol kinase and were normalized to protein content measured by BCA^31^.

Metabolites were analyzed by LC-MS/MS^3^ with small modifications. Worm pellets (~200 mL) were collected in CK14—2 mL lysing kit tubes (Bertin Technologies), flash frozen, and stored at −80 °C. Samples were slowly thawed and homogenized using 4.25 volumes of ice-cold aqueous methanol 98.8%, 2.4 mM ammonium acetate (pH 9), containing 10 mM (^13^C_10_,^15^N_5_)-AMP as internal standard, using bead beating (Precellys plus Cryolys cooler, Bertin Technologies; protocol: 6000 rpm, 2 × 25 s, 15 s pause) and 2 min sonication (cycles of 10 sec on/off, output of 150 W) in a cup-horn sonicator filled with water and ice. Homogenates were incubated 15 min on ice and centrifuged 20,000 *g*, 15 min at 4 °C. Water-soluble metabolites were then extracted from the supernatant by liquid–liquid extraction and analyzed as described^3^. Relative amounts were normalized to protein content using BCA.

### Triglyceride determination

Synchronized 1-day adult animals were harvested and washed with M9 buffer and pellets were flash frozen in liquid N_2_. Pellets were then crushed in mortar and pestle on dry ice and worm powder was resuspended in 300 µl of 5% NP40. Extracts were then collected in tubes, sonicated, then heated at 100 °C for 5 min, and centrifuged at 15,994 × g, 4 °C, for 5 min. Supernatants were used for TG measurements using TG determination kit from Sigma Aldrich according to the manufacturer’s protocol and pellets were resuspended in 0.2 N NaOH and protein amounts were determined using BCA.

### Pharyngeal pumping

Pharyngeal pumping rates were measured by counting the number of grinder movements per 30 s using a stereomicroscope. The worms were always on a lawn of food at rest. Pharyngeal pumping rates were measured in at least ten worms per condition.

### Brood size

Single L4 animals were transferred to fresh agar plates seeded with *E. Coli* OP50 bacteria. The animals were transferred individually to fresh plates every day until they stopped laying eggs after 5–6 days. The progeny number was counted 2 days after egg laying and the brood size was determined by the sum of the number of progeny produced by an individual hermaphrodite.

### Egg laying

Single synchronized 1-day adult animals were transferred to fresh OP50-seeded NGM plates and left for 2 h. Animals were removed from the plates and the number of eggs was counted. We used ten replicates per strain and the experiment was repeated three times.

### Swimming exercise

We used the WMicroTracker machine (Phylum Tech)^77^ to track the swimming activity of the strains. Day 1 adult animals grown on NGM were transferred to 96 well plates containing 100 µl of M9 buffer with 30 animals per well in triplicates, and the swimming behavior was tracked and computed for 10 h.

### Transcriptional reporters imaging and analysis

Transcriptional reporter strains *pgph-2p::GFP* and *pgph-3p::GFP* were obtained from the Dr. Ballie’s library (CGC). Animals were immobilized in 5 mM levamisole and mounted on 2% agarose pads and imaged using the Leica SP5 confocal microscope.

### Statistical analyses

Data are expressed as means ± SEM. Statistical analyses were performed by student’s *t*-test for two groups or one-way ANOVA for multiple groups using Graphpad. For survival curves, we used the Log-rank Mantel–Cox test. Significance is indicated in the figures and legends or included in the supplementary Data.

### Reporting summary

Further information on research design is available in the Nature Research Reporting Summary linked to this article.

## Supplementary information

Supplementary Information

Description of Additional Supplementary Files

Supplementary data 1

Supplementary data 2

Supplementary data 3

Supplementary data 4

Supplementary data 5

Supplementary data 6

Supplementary data 7

Supplementary data 8

Supplementary data 9

Supplementary Movie 1

Supplementary Movie 2

Supplementary Movie 3

Supplementary Movie 4

Supplementary Movie 5

Supplementary Movie 6

Supplementary Movie 7

Supplementary Movie 8

Reporting Summary

## Data Availability

All relevant data generated or analyzed during this study are included in this manuscript and/or its supplementary information. Metabolomics datasets have been deposited at Metabolights^78^ and are available under accession code MTBLS3485. The data underlying Figs. 1–7 and Supplementary Figs. 1–11 are provided as Source data. Any remaining raw data will be available from the corresponding author upon reasonable request. Source data are provided with this paper.

## Source data

Source Data
