# Supplementary material for: Phosphoglycolate phosphatase homologs act as glycerol-3-phosphate phosphatase to control stress and healthspan in C. elegans
Source: Nat Commun. 2022 Jan 11;13:177. doi: 10.1038/s41467-021-27803-6 (PMC8752807; doi:10.1038/s41467-021-27803-6)
Supplement: Supplementary file 1 — Supplementary Information [file 41467_2021_27803_MOESM1_ESM.pdf]

Supplementary information file

Phosphoglycolate phosphatase homologs act as glycerol-3-phosphate phosphatase to control stress and healthspan in *C. elegans*

Possik et al.,

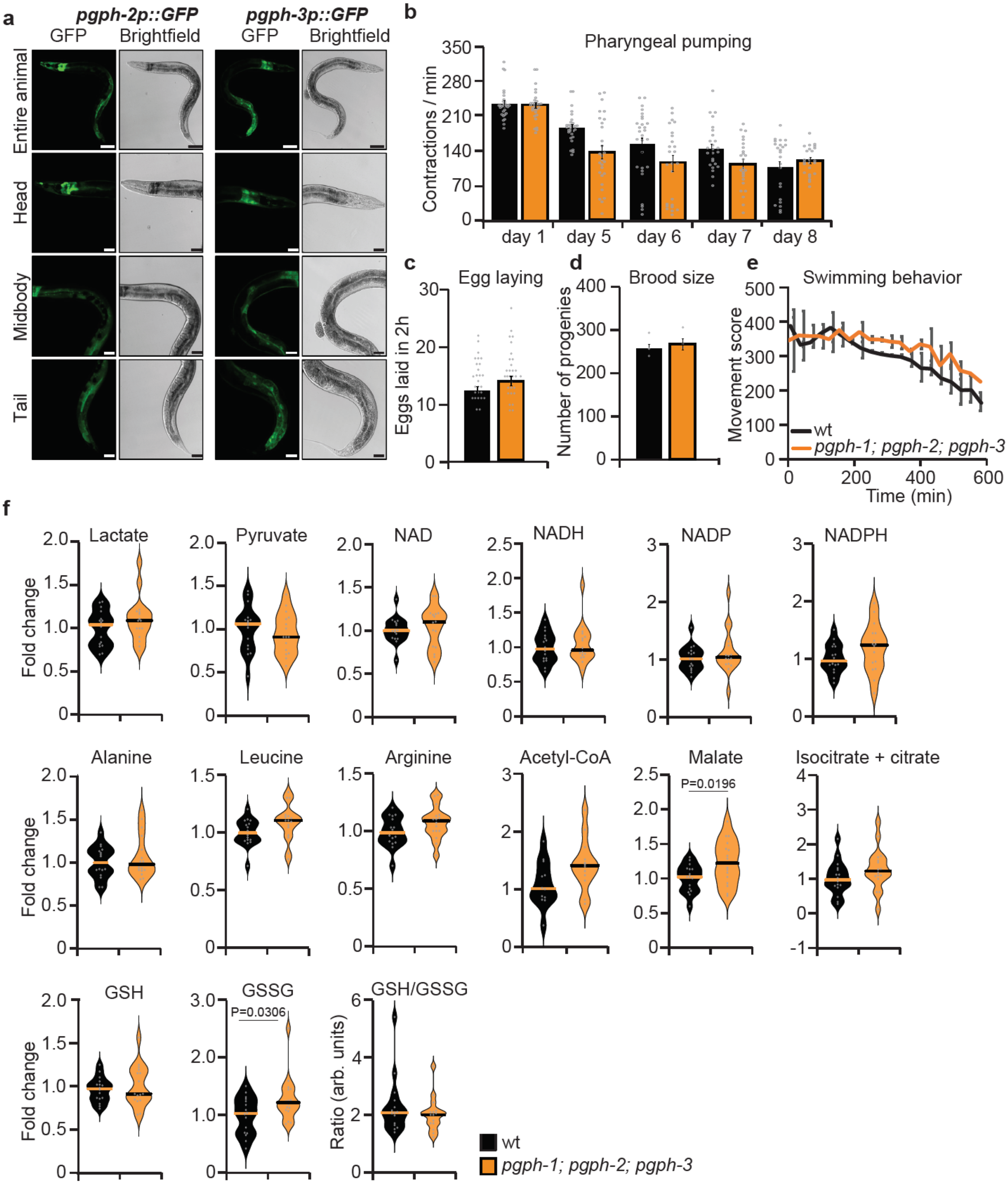
a
pgph-2p::GFP
pgph-3p::GFP
GFP
Brightfield
Entire animal
Head
Midbody
Tail
b
Pharyngeal pumping
Contractions / min
day 1
day 5
day 6
day 7
day 8
c
Egg laying
Eggs laid in 2h
d
Brood size
Number of progenies
e
Swimming behavior
Movement score
Time (min)
wt
pgph-1; pgph-2; pgph-3
f
Lactate
Pyruvate
NAD
NADH
NADP
NADPH
Alanine
Leucine
Arginine
Acetyl-CoA
Malate
P=0.0196
Isocitrate + citrate
GSH
GSSG
P=0.0306
GSH/GSSG
Fold change
Ratio (arb. units)
wt
pgph-1; pgph-2; pgph-3

Supplementary Fig. 1 related to figure 1. Physiological and metabolic characterization of the *pgph-1; pgph-2; pgph-3* mutant. a Representative confocal images of *pgph-2p::GFP* and *pgph-3p::GFP* transcriptional strains. Scale bars represent 100 µm. **b-e** Characterization of the WT and *pgph-1; pgph-2; pgph-3* mutant animals, with pharyngeal pumping rates (**b**), egg laying behavior for 2 h (**c**), brood size (**d**) and swimming activity for 10 h (**e**). For panels b, c, and d 10 animals have been used per group per independent repeat and the experiment has been repeated three independent times. The non-significant changes have been obtained using Two-way ANOVA with Bonferroni correction **(b)** and unpaired two-tailed student's t test **(c).** For panel **d**, Data represent mean ± SEM from 3 independent experiments. For e, duplicates of 50 animals per group per independent repeat have been used and the experiment has been performed two independent times. The non-significant changes have been obtained using Two-way ANOVA with Bonferroni correction. **f** Violin plots showing metabolite levels in addition to those shown in Fig. 1c of synchronized L4/young adult *pgph-1; pgph-2; pgph-3* mutant strain in comparison to WT animals grown on NGM plates. Lines in violin plots denote the median of the analyzed groups and gray circles indicate individual data points (from 4 biological replicates per group per independent experiment and 2 independent repeats). P values are obtained using two-tailed student's t test. Significance in all figures: ****P<0.0001. Data are provided as Source Data.

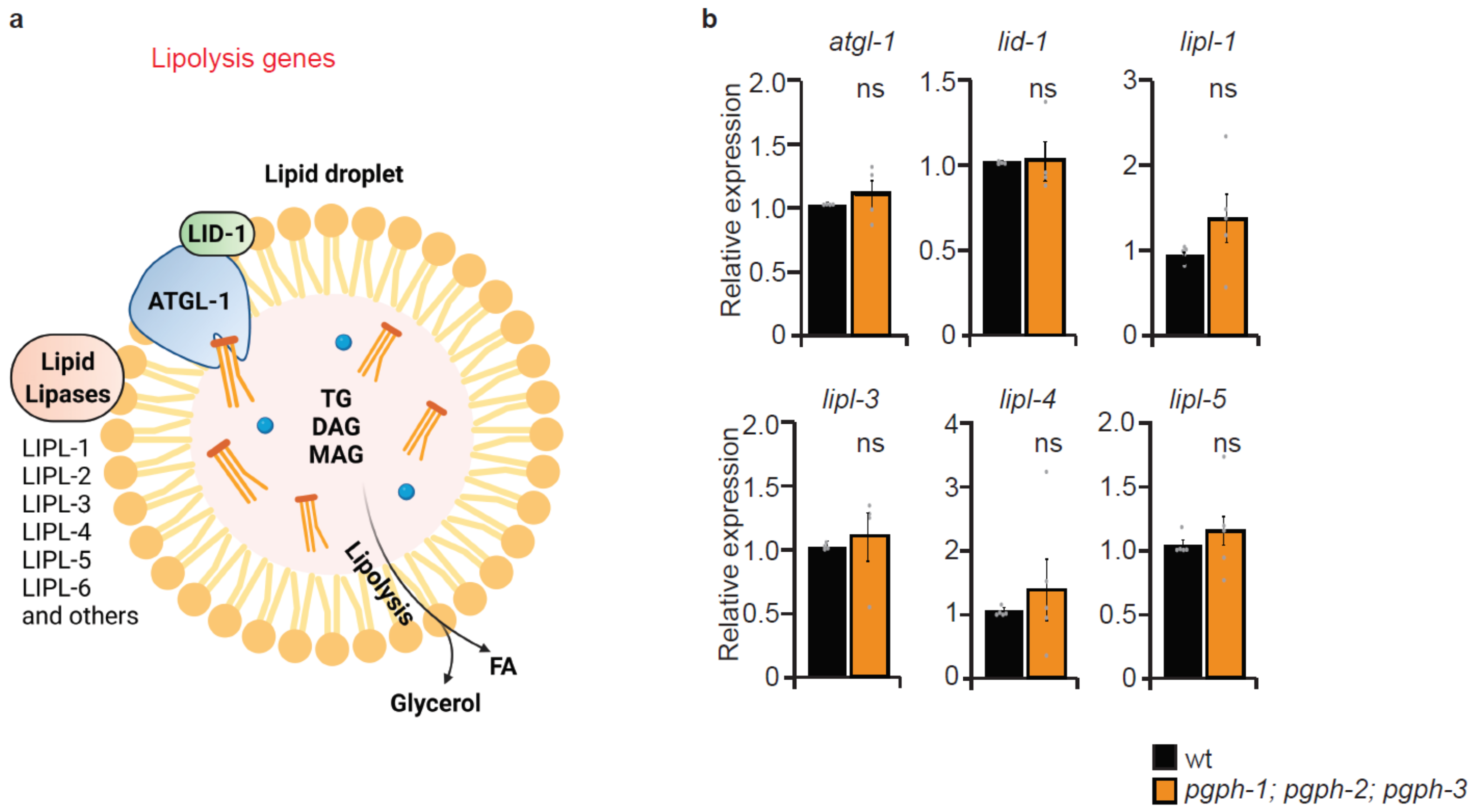

Supplementary Fig. 2 related to figure 2. Lipase genes are not significantly altered by PGPH deletion. a Simplified scheme illustrating lipolysis in *C. elegans*. Scheme was generated using Biorender. **b** Relative expression of indicated genes in WT animals and *pgph-1; pgph-2; pgph-3* synchronized young adult animals. Data represent mean ± SEM, n=4 independent experiments. P values are obtained using unpaired two-tailed student's t test. Data are provided as Source Data.

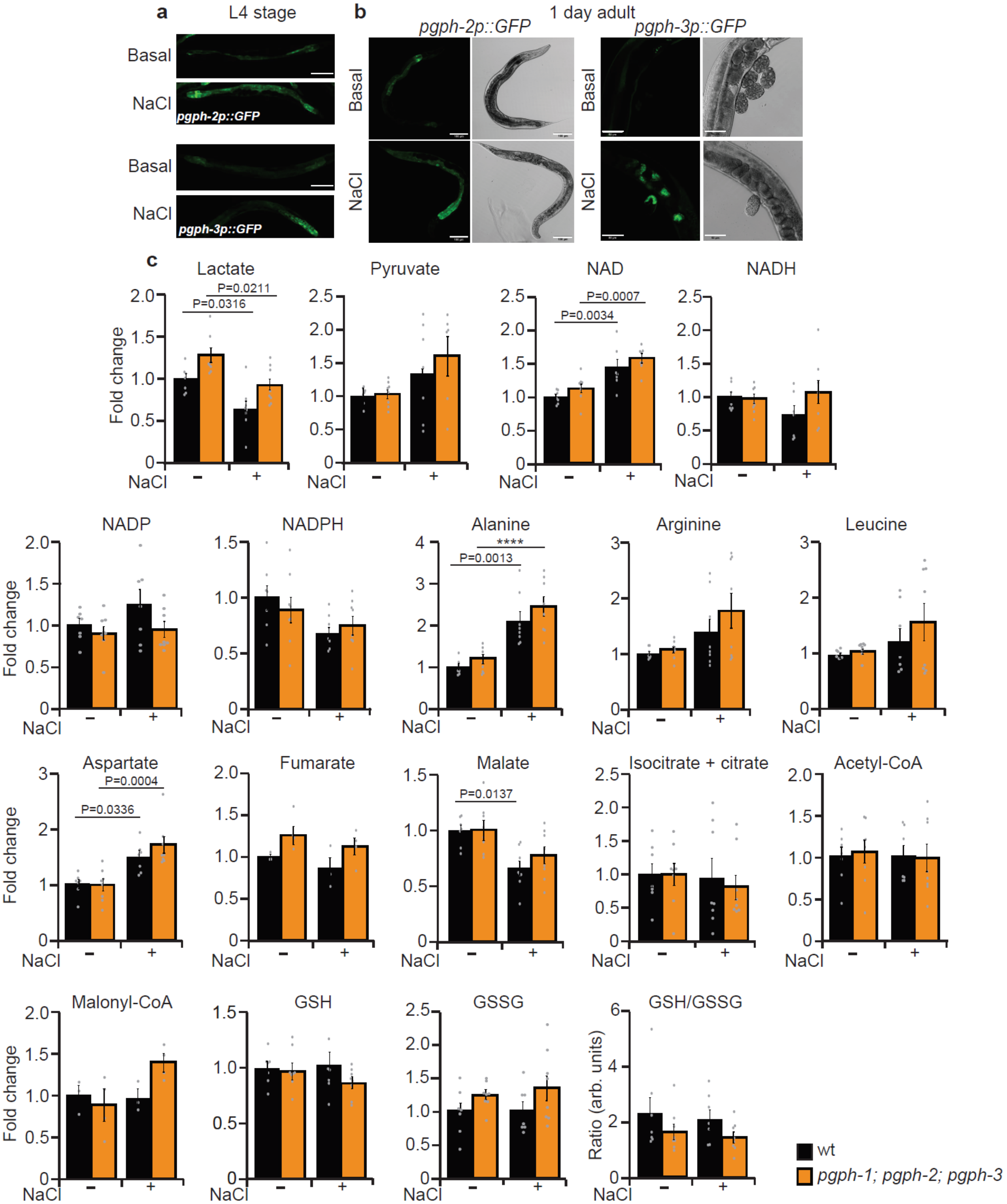

a
L4 stage
Basal
NaCl
pgph-2p::GFP
Basal
NaCl
pgph-3p::GFP
b
1 day adult
pgph-2p::GFP
pgph-3p::GFP
Basal
NaCl
c
Lactate
P=0.0211
P=0.0316
Pyruvate
NAD
P=0.0007
P=0.0034
NADH
Fold change
NaCl
NADP
NADPH
Alanine
****
P=0.0013
Arginine
Leucine
Aspartate
P=0.0004
P=0.0336
Fumarate
Malate
P=0.0137
Isocitrate + citrate
Acetyl-CoA
Malonyl-CoA
GSH
GSSG
GSH/GSSG
Ratio (arb. units)
wt
pgph-1; pgph-2; pgph-3

Supplementary Fig. 3 related to figure 3. PGPH tissue-specific expression and metabolic profiles of triple *pgph* mutant animals following salt stress. a-b Representative confocal images of *pgph-2p::GFP* and *pgph-3p::GFP* transcriptional reporter strains at basal level or after exposure to 400 mM NaCl for 2 h. **a** L4 animals. Scale bars represent 150 µm. **b** 1 day adult animals. Scale bars represent 100 µm for *pgph-2p::GFP* and 50 µm for *pgph-3p::GFP*. The confocal imaging has been performed three independent times and best representative images have been chosen for publication. **c** Metabolite levels in addition to those shown in Fig. 3c in synchronized L4/young adult WT and *pgph-1; pgph-2; pgph-3* animals at basal level or after exposure to 400 mM NaCl for 2 h. Data represent mean ± SEM, n=8 biological replicates from 2 independent experiments. P values are obtained using one-way ANOVA with Bonferroni test. Data are provided as Source Data.

a

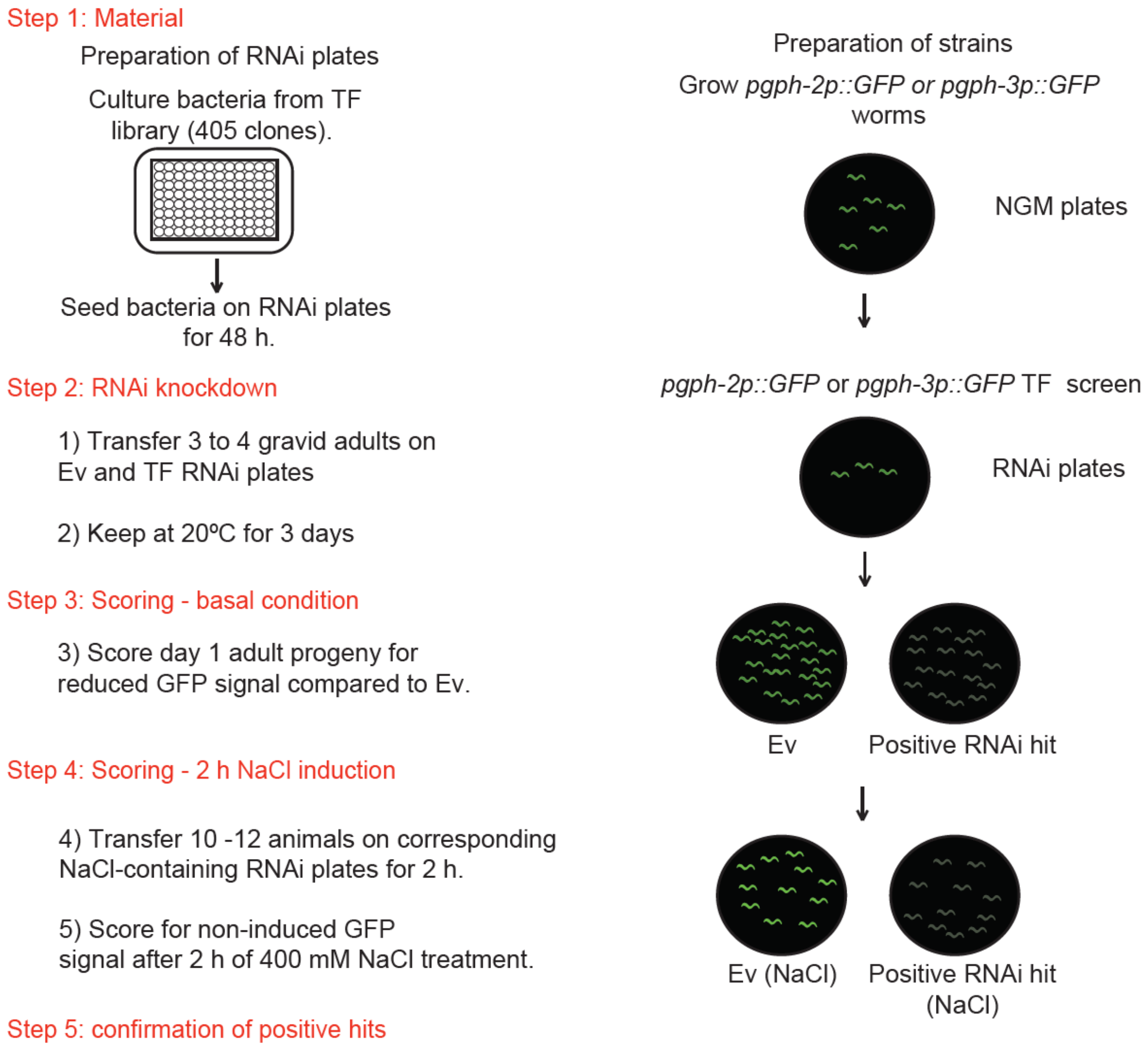

b

Gene ID	Gene name	Gene description
B0286.5	*fkh-6*	Forkhead transcription factor family
C33D3.1	*elt-2*	Transcription factor *elt-2*
C42D8.4	*ets-5*	ETS class transcription factor
D1081.2	*unc-120*	Uncoordinated 120
E03H4.13	*nhr-89*	Nuclear hormone receptor family member *nhr-89*
F54F7.1	*taf-7.1*	TAF (TBP-associated transcription factor) family
R13H8.1	*daf-16*	Forkhead box protein O

c

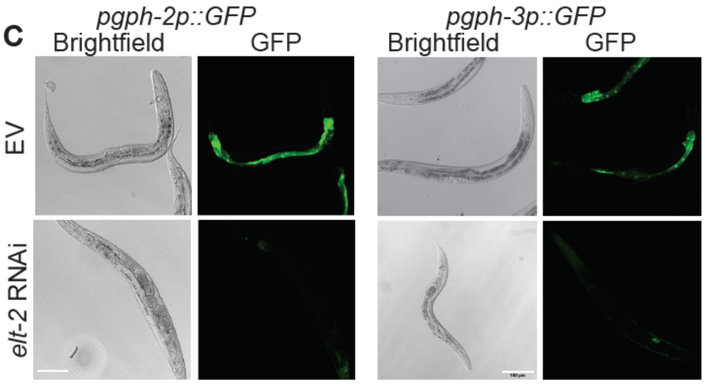

Supplementary Fig. 4: Transcription factor RNAi screen of regulators of *pgph-2* and *pgph-3* genes following 2 h salt stress. a Representative scheme describing the screen. **b** List of transcription factors identified common to *pgph-2* and *pgph-3*. **c** Confocal fluorescence and brightfield imaging of *pgph-2* and *pgph-3* transcriptional reporter lines at basal level treated with EV or *elt-2* RNAi. Scale bars represent 150 µm. This experiment has been repeat 3 independent times and 10 worms per group have been imaged every independent repeat. The detailed list of the identified transcription factors is shown in Supplementary Data 7.

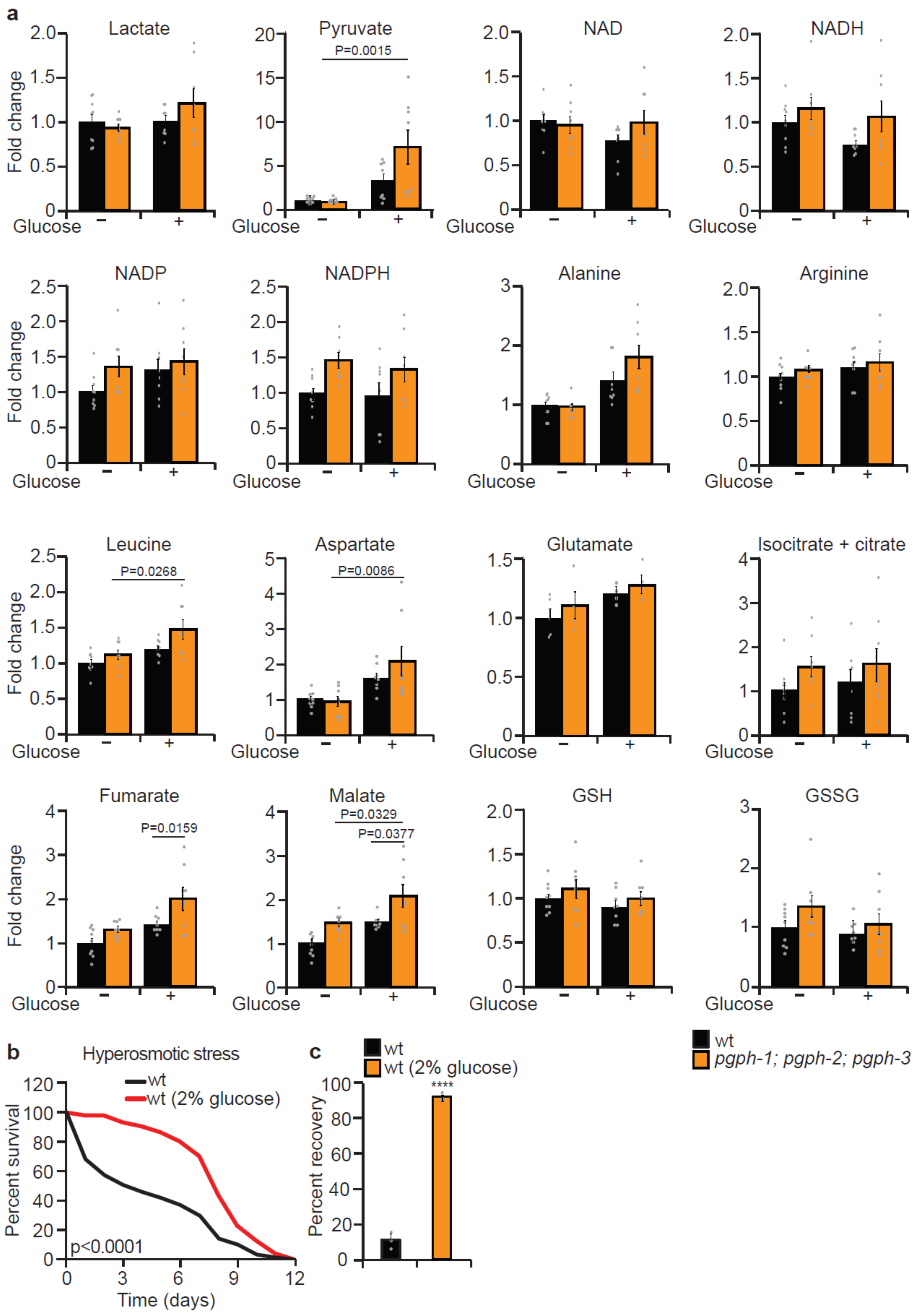

a
Lactate
Pyruvate
P=0.0015
NAD
NADH
NADP
NADPH
Alanine
Arginine
Leucine
P=0.0268
Aspartate
P=0.0086
Glutamate
Isocitrate + citrate
Fumarate
P=0.0159
Malate
P=0.0329
P=0.0377
GSH
GSSG
Fold change
Glucose
–
+
wt
pgph-1; pgph-2; pgph-3
b
Hyperosmotic stress
wt
wt (2% glucose)
Percent survival
p<0.0001
Time (days)
c
wt
wt (2% glucose)
Percent recovery
****

Supplementary Fig. 5 related to figure 4: Metabolic profiles of triple *pgph* mutant animals following excess glucose. a Metabolite levels in addition to those shown in Fig. 4c in synchronized L4/young adult WT and *pgph-1; pgph-2; pgph-3* animals grown on NGM plates or plates supplemented with 2% glucose. Data represent mean ± SEM, n=8 replicates from 2 independent experiments. Significance in all figures: ****$P<0.0001$. P values are obtained by one-way ANOVA with Bonferroni test. **b, c** Percent survival (**b**) and recovery (**c**) of 1 day adult WT animals pretreated or not with 2% glucose and exposed to 400 mM NaCl. For survival experiments, the number of separate experiments, animals and detailed statistics are shown in Supplementary Data 1. The P value of the survival curve is obtained using two-sided Mantel-Cox test while the P value of the recovery assay is obtained using two-tailed t test. For the salt stress recovery assay, the experiment has been repeated 3 independent times (n= 272 for the control group, n=244 for the glucose treated group). Data are provided as Source Data.

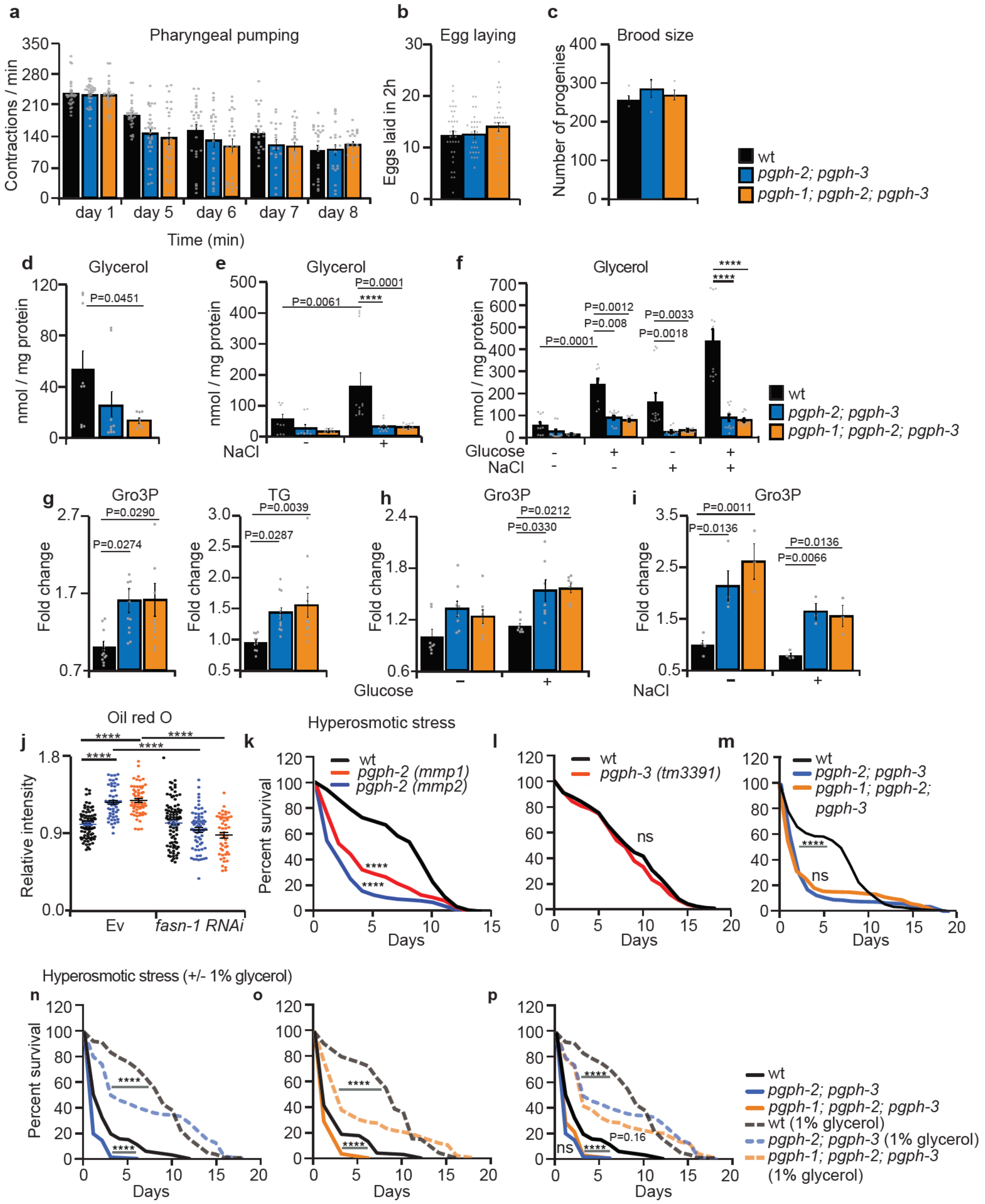

a
Pharyngeal pumping
Contractions / min
350
280
210
140
70
0
day 1
day 5
day 6
day 7
day 8
Time (min)
b
Egg laying
Eggs laid in 2h
30
20
10
0
c
Brood size
Number of progenies
400
300
200
100
0
wt
pgph-2; pgph-3
pgph-1; pgph-2; pgph-3
d
Glycerol
nmol / mg protein
120
80
40
0
P=0.0451
e
Glycerol
nmol / mg protein
500
400
300
200
100
0
P=0.0061
P=0.0001
****
NaCl
-
+
f
Glycerol
nmol / mg protein
700
600
500
400
300
200
100
0
P=0.0001
P=0.0012
P=0.008
P=0.0033
P=0.0018
****
****
Glucose
NaCl
wt
pgph-2; pgph-3
pgph-1; pgph-2; pgph-3
g
Gro3P
Fold change
2.7
1.7
0.7
P=0.0290
P=0.0274
TG
3.0
2.5
2.0
1.5
1.0
0.5
P=0.0039
P=0.0287
h
Gro3P
Fold change
2.4
1.8
1.2
0.6
P=0.0212
P=0.0330
Glucose
–
+
i
Gro3P
Fold change
3.5
2.5
1.5
0.5
P=0.0011
P=0.0136
P=0.0136
P=0.0066
NaCl
–
+
j
Oil red O
Relative intensity
1.8
0.9
0.0
****
****
****
****
Ev
fasn-1 RNAi
Hyperosmotic stress
k
Percent survival
120
100
80
60
40
20
0
wt
pgph-2 (mmp1)
pgph-2 (mmp2)
****
****
0
5
10
15
Days
l
wt
pgph-3 (tm3391)
ns
0
5
10
15
20
Days
m
wt
pgph-2; pgph-3
pgph-1; pgph-2; pgph-3
****
ns
Days
Hyperosmotic stress (+/- 1% glycerol)
n
Percent survival
****
****
Days
o
****
****
Days
p
****
****
ns
P=0.16
Days
wt
pgph-2; pgph-3
pgph-1; pgph-2; pgph-3
wt (1% glycerol)
pgph-2; pgph-3 (1% glycerol)
pgph-1; pgph-2; pgph-3
(1% glycerol)

Supplementary Fig 6: PGPH-2 is the primary PGPH isozyme contributing to Gro3P hydrolysis. a-c Characterization of the WT, *pgph-2; pgph-3* and *pgph-1; pgph-2; pgph-3* mutant animals with pharyngeal pumping rates (**a**), egg laying (**b**) and brood size (**c**). Data represent mean ± SEM. A total of 10 animals have been used per group per independent repeat and the experiments have been repeated 3 independent times. **d-f** Glycerol content in WT, *pgph-2; pgph-3* and *pgph-1; pgph-2; pgph-3* synchronized L4/young adult animals grown on: NGM plates (**d**), NGM plates treated or not with 400mM NaCl for 3 hr (**e**), NGM plates or plates supplemented with 2% glucose **(f)**. Data represent mean ± SEM, 3 biological replicates per group per independent repeat and 3 independent experiments. **g** Relative Gro3P and TG levels of WT, *pgph-2; pgph-3* and *pgph-1; pgph-2; pgph-3* mutant synchronized L4/young adults grown on NGM plates. Data represent mean ± SEM. For Gro3P (4 biological replicates per group per independent repeat and 3 independent experiments). For TG measurements (3 biological replicates per group per independent repeat and 3 independent experiments). **h-i**, Relative Gro3P levels of WT, *pgph-2; pgph-3* and *pgph-1; pgph-2; pgph-3* mutant synchronized L4/young adults grown on NGM plates or plates supplemented with 2% glucose (**h**), or plates with 400 mM NaCl (**i**). Protocol detailed in methods. Data represent mean ± SEM, 2 independent experiments, n=8 (**h), n**=4 **(i**). **j** Oil red O staining and quantification of WT, *pgph-2; pgph-3*, and *pgph-1; pgph-2; pgph-3* mutant strains treated or not with Ev and *fasn-1* RNAi. Sample numbers: n=76 (WT-Ev), n=53 (*pgph-1; pgph-2*-Ev), n=59 (*pgph-1; pgph-2; pgph-3*-Ev) and n=93 (WT-*fasn-1* RNAi), n=68 (*pgph-1; pgph-2-fasn-1* RNAi), n=50 (*pgph-1; pgph-2; pgph-3-fasn-1* RNAi). Data represent mean ± SEM from 2 independent experiments. **k-m** Survival curves of indicated strains exposed to 400 mM NaCl. **n-p** Survival curves of indicated strains grown on plates supplemented or not with 1% glycerol exposed to 400 mM NaCl. Number of separate experiments, animals and detailed statistics are shown in Supplementary Data 1. For experiments **a-j**, P values are obtained by one-way ANOVA using Bonferroni test. For survival curves **k-p**, P values are obtained using two-sided Mantel-Cox test. ****P<0.0001. Data are provided as Source Data.

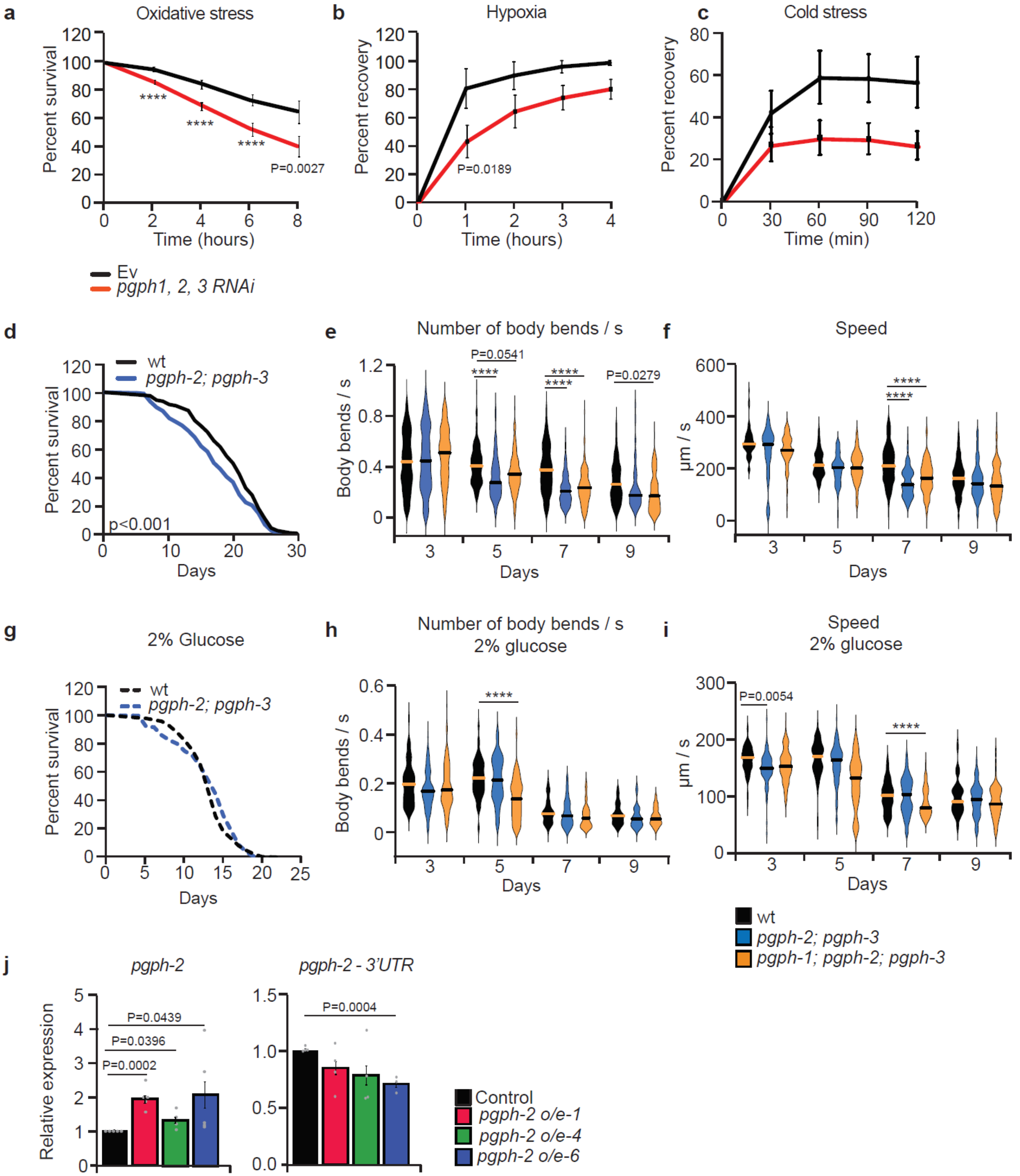
a
Oxidative stress
Percent survival
Time (hours)
****
****
****
P=0.0027
b
Hypoxia
Percent recovery
Time (hours)
P=0.0189
c
Cold stress
Percent recovery
Time (min)
Ev
pgph1, 2, 3 RNAi
d
wt
pgph-2; pgph-3
Percent survival
p<0.001
Days
e
Number of body bends / s
Body bends / s
P=0.0541
****
****
****
P=0.0279
Days
f
Speed
µm / s
****
****
Days
g
2% Glucose
wt
pgph-2; pgph-3
Percent survival
Days
h
Number of body bends / s
2% glucose
Body bends / s
****
Days
i
Speed
2% glucose
µm / s
P=0.0054
****
Days
wt
pgph-2; pgph-3
pgph-1; pgph-2; pgph-3
j
pgph-2
Relative expression
P=0.0439
P=0.0396
P=0.0002
pgph-2 - 3'UTR
P=0.0004
Control
pgph-2 o/e-1
pgph-2 o/e-4
pgph-2 o/e-6

Supplementary Fig. 7 related to figure 5. PGPH loss decreases stress response and lifespan and healthspan in normal and high glucose conditions. a Percent survival of L4/young adult WT animals treated with Ev or *pgph* RNAi to 100 mM paraquat. Percent recovery of 1 day adult animals treated with Ev or *pgph* RNAi after 24 hours hypoxia (**b**) and 4°C cold stress for 16 h (**c**). The number of separate experiments, animals and detailed statistics are shown in Supplementary Data 4-6. P values are obtained using two-way ANOVA with Bonferroni test. **d** Lifespan survival curves of indicated worm strains grown on NGM plates. P values are obtained by two-sided Mantel-Cox test. **e-f** Violin plots showing locomotion analysis including the number of body bends per second (**e**) and speed (**f**) in WT, *pgph-2; pgph-3, and pgph-1; pgph-2; pgph-3* mutant animals grown on NGM plates in comparison to control nematodes at days 3, 5, 7 and 9 of age. The median is indicated by a line in the plot and exact numbers are shown in data source. The experiment has been repeated 3 independent times. P values are obtained using one-way ANOVA with Bonferroni test. **g** Lifespan survival curves of indicated worm strains grown on plates supplemented with 2% glucose. P values are obtained by two-sided Mantel-Cox test. **h-i** Locomotion analysis including the number of body bends per second (**h**) and speed (**i**) in WT, *pgph-2; pgph-3* and *pgph-1; pgph-2; pgph-3* mutant animals grown on plates supplemented with 2% glucose in comparison to control nematodes at days 3, 5, 7 and 9 of age. The median is indicated by a line in the plot and exact numbers are shown in data source. The experiment has been repeated 3 independent times. P values are obtained using one-way ANOVA with Bonferroni test. For lifespan and glucotoxicity curves, the number of separate experiments, animals and detailed statistics are shown in Supplementary Data 2 and 3. **j** Relative expression of overexpressed *pgph-2* and endogenous *pgph-2* in indicated *pgph-2* transgenic overexpressing animals in comparison to control nematodes. Data represent mean ± SEM, 3 biological replicates and 3 independent experiments. P values are obtained using one-way ANOVA with Bonferroni test. Significance in all figures: ****P<0.0001. Data are provided as Source Data.

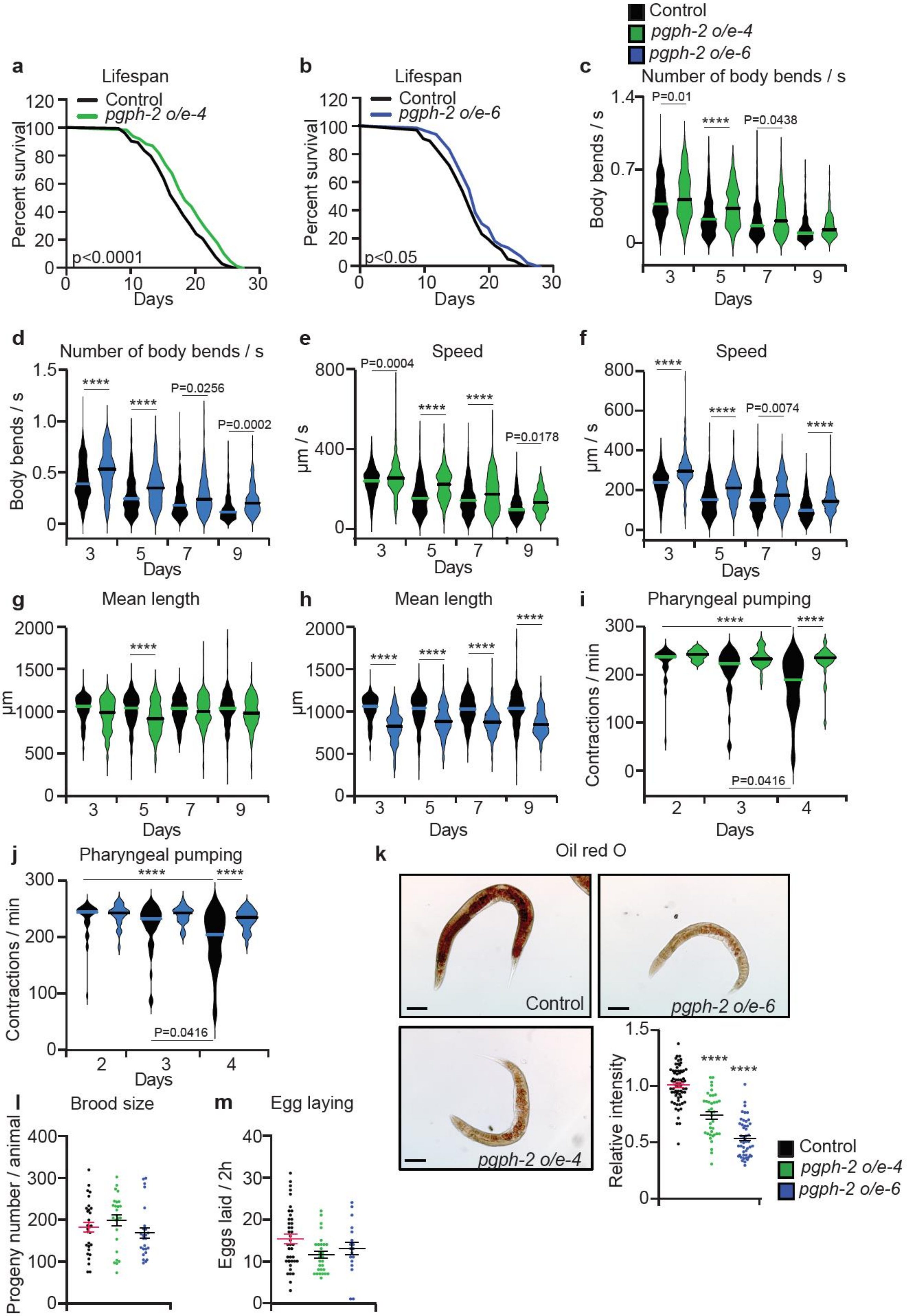

Control
pgph-2 o/e-4
pgph-2 o/e-6
a
Lifespan
Control
pgph-2 o/e-4
Percent survival
p<0.0001
Days
b
Lifespan
Control
pgph-2 o/e-6
Percent survival
p<0.05
Days
c
Number of body bends / s
Body bends / s
P=0.01
****
P=0.0438
Days
d
Number of body bends / s
Body bends / s
****
****
P=0.0256
P=0.0002
Days
e
Speed
µm / s
P=0.0004
****
****
P=0.0178
Days
f
Speed
µm / s
****
****
P=0.0074
****
Days
g
Mean length
µm
****
Days
h
Mean length
µm
****
****
****
****
Days
i
Pharyngeal pumping
Contractions / min
****
****
P=0.0416
Days
j
Pharyngeal pumping
Contractions / min
****
****
P=0.0416
Days
k
Oil red O
Control
pgph-2 o/e-6
pgph-2 o/e-4
Relative intensity
****
****
l
Brood size
Progeny number / animal
m
Egg laying
Eggs laid / 2h

Supplementary Fig. 8 related to figure 6. PGPH-2 overexpression improves healthspan and decreases fat content. a-b Lifespan of indicated *pgph-2* transgenic overexpressing lines in comparison to control nematodes. Number of separate experiments, animals and detailed statistics are shown in Supplementary Data 2. P values are obtained using two-sided Mantel-Cox test. **c-h** Violin plots showing locomotion and body size analyses including the number of body bends per second (**c-d**), speed (**e-f**), mean length (**g-h**) in *pgph-2* overexpressing animals in comparison to control nematodes at days 3, 5, 7 and 9 of age. The median is indicated by a line in the plot and more than 150 tracks per group have been analyzed. Exact numbers are shown in data source. The experiment has been repeated 3 independent times. P values are obtained using one-way ANOVA with Bonferroni test. **i-j** Violin plots showing pharyngeal pumping rates of control and indicated *pgph-2* overexpressing transgenic lines at days 2, 3, and 4 of age. Sample numbers: n= 35 (control day 2), n= 26 (*pgph-2 o/e4* day 2), n= 37 (control day 3), n= 31 (*pgph-2 o/e4* day 3), n= 36 (control day 4), n= 31 (*pgph-2 o/e4* day 4), n= 27 (*pgph-2 o/e6* day 2), n=31 (*pgph-2 o/e6* day 3), n=30 (*pgph-2 o/e6* day 4). The median is indicated by a line in the plots and experiment has been repeated 3 independent times. **k** Oil red O staining and quantification in 3 days old adult control and indicated *pgph-2* overexpressing transgenic nematodes. Scale bars represent 50 µm. Sample numbers: n= 66 (control), n= 38 (*pgph-2 o/e4*), n= 48 (*pgph-2 o/e6*). Data represent mean ± SEM from 3 independent experiments. **l-m** Brood size (**l**) and egg laying (**m**) in *pgph-2* overexpressing animals in comparison to control nematodes. Data represent mean ± SEM, n=10 per group per independent repeat and 3 independent experiments. P values from Figs **c-m** are obtained by one-way ANOVA with Bonferroni test. Significance in all figures: ****P<0.0001. Data are provided as Source Data.

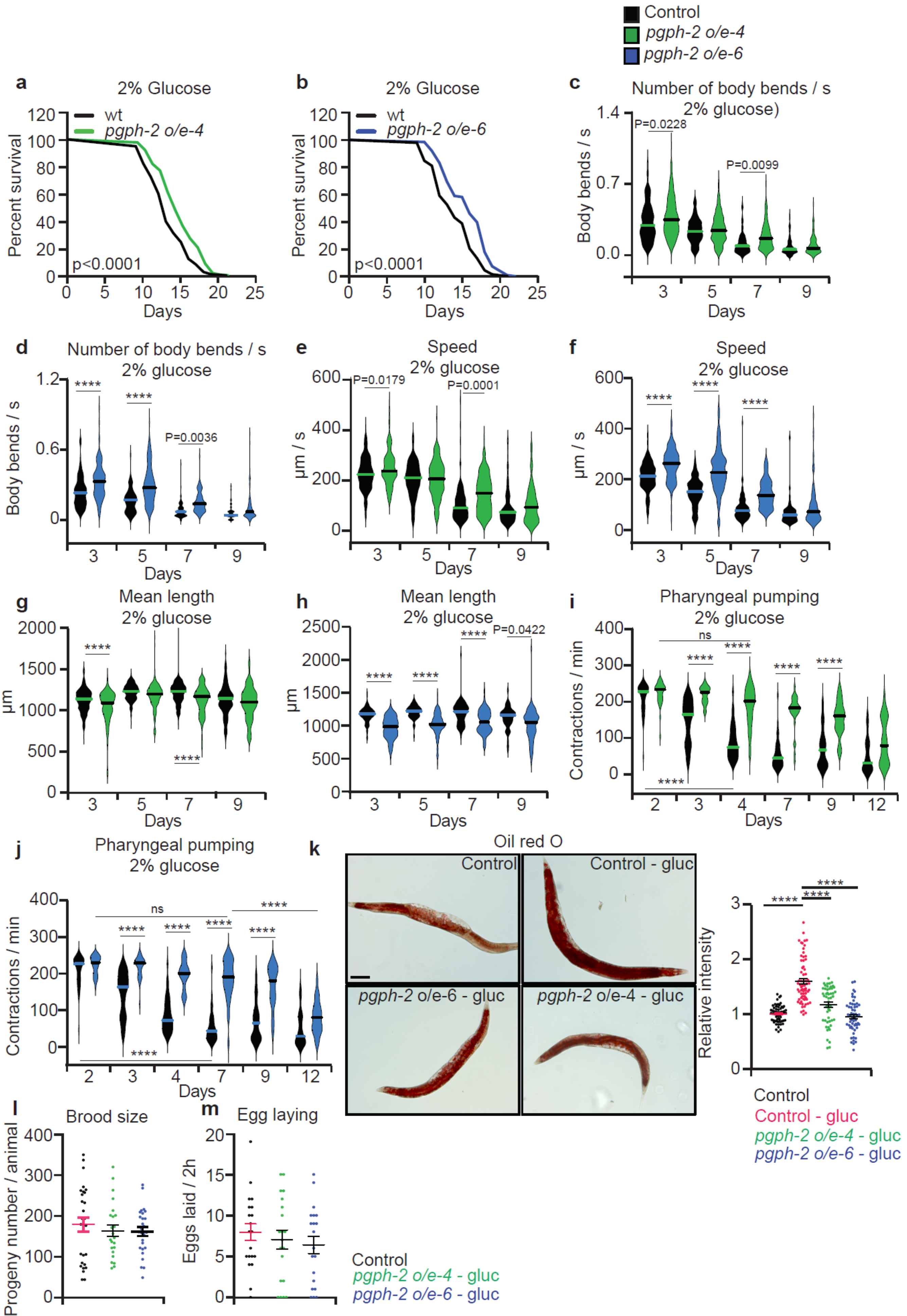

Control
pgph-2 o/e-4
pgph-2 o/e-6
a
2% Glucose
wt
pgph-2 o/e-4
Percent survival
p<0.0001
Days
b
2% Glucose
wt
pgph-2 o/e-6
Percent survival
p<0.0001
Days
c
Number of body bends / s
2% glucose)
Body bends / s
P=0.0228
P=0.0099
Days
d
Number of body bends / s
2% glucose
Body bends / s
P=0.0036
Days
e
Speed
2% glucose
µm / s
P=0.0179
P=0.0001
Days
f
Speed
2% glucose
µm / s
Days
g
Mean length
2% glucose
µm
Days
h
Mean length
2% glucose
µm
P=0.0422
Days
i
Pharyngeal pumping
2% glucose
Contractions / min
ns
Days
j
Pharyngeal pumping
2% glucose
Contractions / min
ns
Days
k
Oil red O
Control
Control - gluc
pgph-2 o/e-6 - gluc
pgph-2 o/e-4 - gluc
Relative intensity
Control
Control - gluc
pgph-2 o/e-4 - gluc
pgph-2 o/e-6 - gluc
l
Brood size
Progeny number / animal
m
Egg laying
Eggs laid / 2h
Control
pgph-2 o/e-4 - gluc
pgph-2 o/e-6 - gluc

Supplementary Fig. 9 related to figure 7. PGPH-2 overexpression increases lifespan under excess glucose and reduces glucose-induced fat deposition. a-b Survival curves in indicated *pgph-2* transgenic overexpressing lines in comparison to control nematodes in the presence of 2% glucose. Number of separate experiments, animals and detailed statistics are shown in Supplementary Data 3. P values are obtained using two-sided Mantel-Cox test. **c-h** Violin plots showing locomotion and body size analyses including the number of body bends per second (**c-d**), speed (**e-f**), mean length (**g-h**) in indicated *pgph-2* overexpressing animals grown on plates supplemented with 2% glucose in comparison to control nematodes at days 3, 5, 7 and 9 of age. The median is indicated by a line in the plot and around 150 tracks per group have been analyzed. Exact numbers are shown in data source. The experiment has been repeated 3 independent times. **i-j** Violin plots showing pharyngeal pumping rates of control and indicated *pgph-2* overexpressing transgenic lines at indicated days in the presence of 2% glucose. Lines in the plots denote median rates. Sample numbers: n= 36 (control day 2), n= 34 (control day 3), n=30 (control days 4, 7, 9, 12), n= 30 (*pgph-2 o/e4 and pgph-2 o/e6* days 2, 3, 4, 7, 9, 12). **k** Oil red O staining and quantification in 3 days old adult control and indicated *pgph-2* overexpressing transgenic nematodes grown on plates supplemented with 2% glucose. Scale bars represent 50 µm. Data represent mean ± SEM from 3 independent experiments. Sample numbers: n= 65 (control), n= 68 (control-2% glucose), n= 51 (*pgph-2 o/e4* - 2% glucose), n= 55 (*pgph-2 o/e6* - 2% glucose).**l-m** Brood size (**l**) and egg laying (**m**) in indicated *pgph-2* overexpressing animals grown on plates supplemented with 2% glucose in comparison to control nematodes. Data represent mean ± SEM, n=10 per group per independent repeat and 3 independent experiments. P values of Figs **c-m** are obtained by one-way ANOVA with Bonferroni test. Significance in all figures: ****P<0.0001. Data are provided as Source Data.

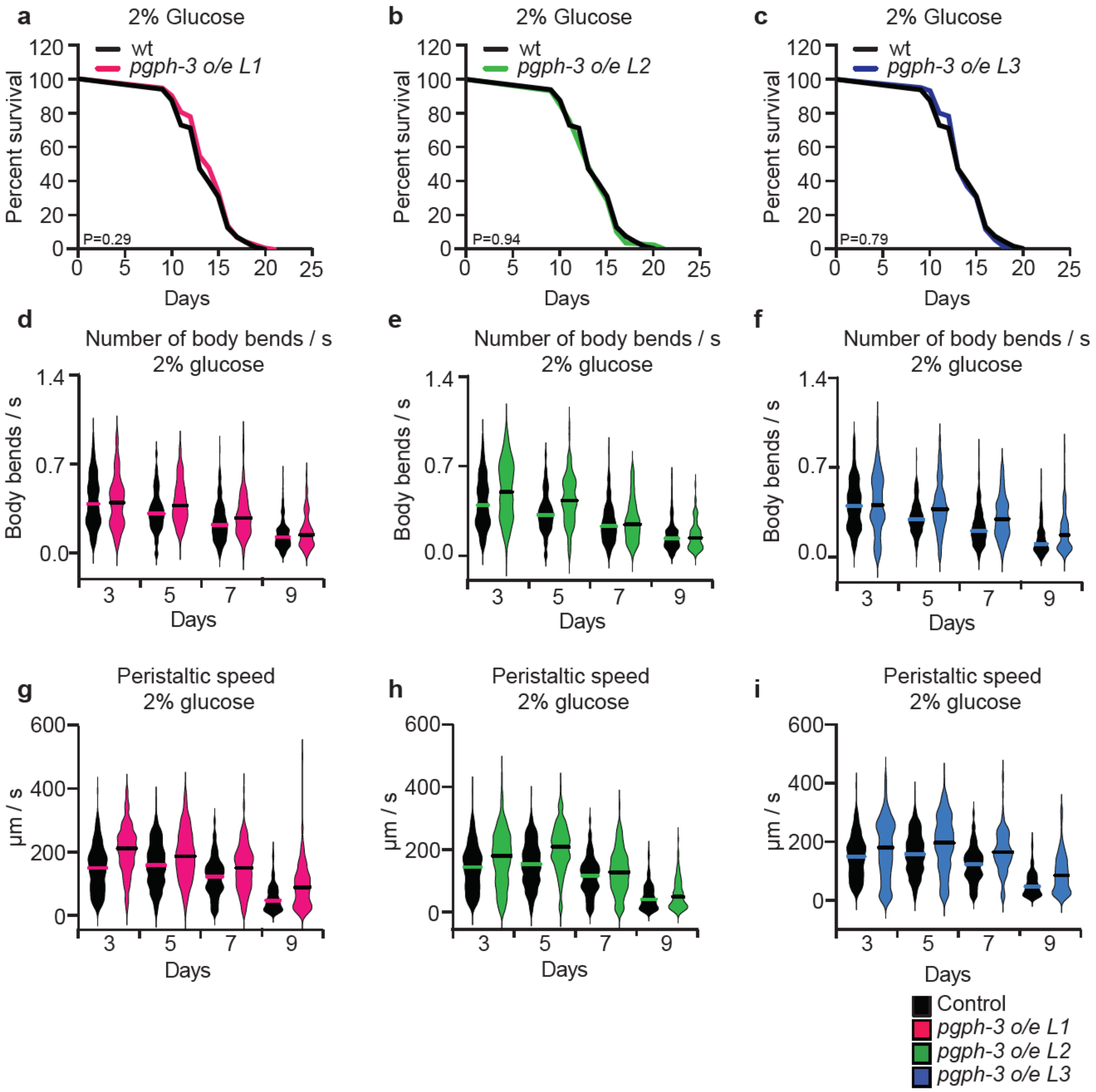

Supplementary Fig. 10. PGPH-3 overexpression has no effect on lifespan and healthspan in the presence of glucose. a-c Survival curves in the presence of 2% glucose in indicated *pgph-3* transgenic overexpressing lines in comparison to control nematodes. Number of separate experiments, animals and detailed statistics are shown in Supplementary Data 3. P values are obtained using two-sided Mantel-Cox test. **d-i** Violin plots showing locomotion analyses including

the number of body bends per second (**d-f**) and peristaltic speed (**g-i**) in indicated *pgph-3* overexpressing animals grown on NGM plates or plates supplemented with 2% glucose in comparison to control nematodes at days 3, 5, 7 and 9 of age The median is indicated by a line in the plot and around 150 tracks per group have been analyzed. Exact numbers are shown in data source. These experiments have been repeated 3 independent times. P values are obtained using one-way ANOVA with Bonferroni test. Data are provided as Source Data.

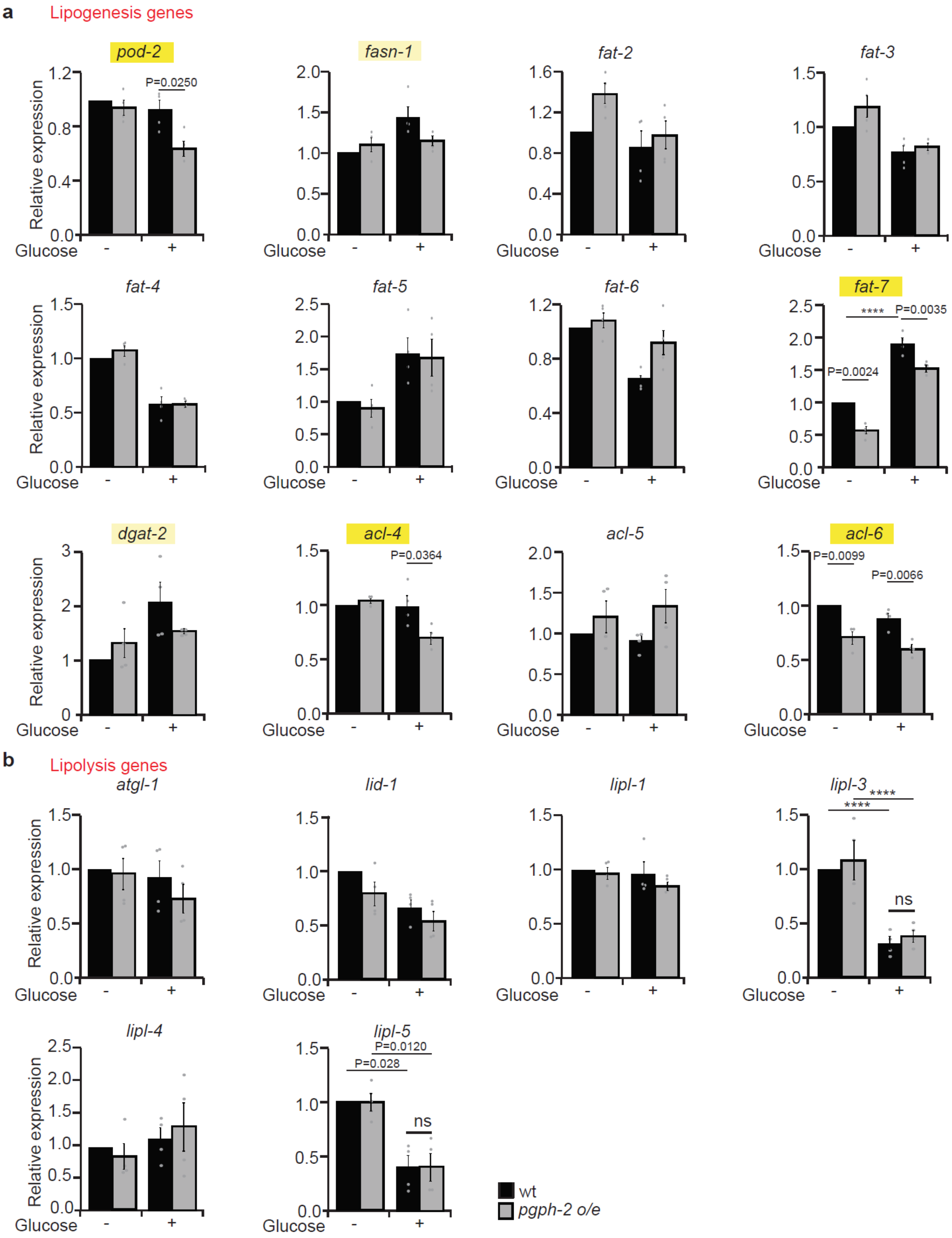
a
Lipogenesis genes
pod-2
fasn-1
fat-2
fat-3
fat-4
fat-5
fat-6
fat-7
dgat-2
acl-4
acl-5
acl-6
P=0.0250
P=0.0024
P=0.0035
P=0.0364
P=0.0099
P=0.0066
Relative expression
Glucose
b
Lipolysis genes
atgl-1
lid-1
lipl-1
lipl-3
lipl-4
lipl-5
ns
P=0.0120
P=0.028
wt
pgph-2 o/e

Supplementary Fig. 11. PGPH-2 o/e decreases the expression of several lipogenesis genes without affecting the expression of lipolysis genes. a-b Relative expression of indicated genes in control animals and *pgph-2-oe* treated or not with 2% glucose. Data represent mean ± SEM, 2 biological replicates and 2 independent experiments. P values are obtained using two-tailed student's t test. Significance in all figures: ****P<0.0001. Data are provided as Source Data.

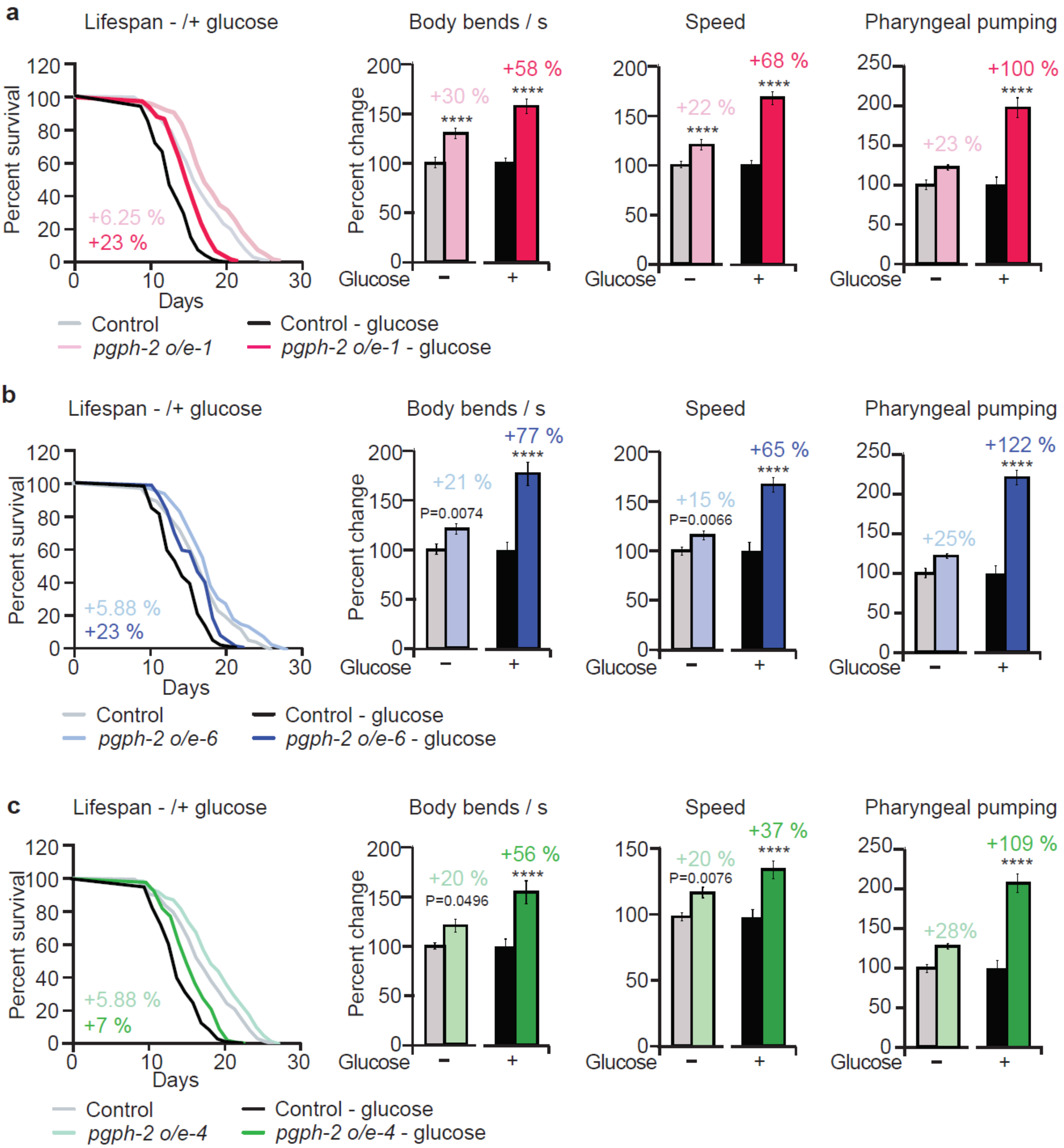

Supplementary Fig. 12. Comparison of lifespan and healthspan effects in *pgph-2 o/e* lines in the presence and absence of glucose. **a-c** Lifespan and healthspan parameters in the presence

and absence of 2% glucose in three separate *pgph-2* o/e lines, *pgph-2 o/e1* (a), *pgph-2 o/e6* (b), *pgph-2 o/e 4* (c). For the survival curves, the number of separate experiments, animals and detailed statistics are shown in Supplementary Data 2 and 3. Percent change in median survival is indicated. P values are obtained using two-sided Mantel-Cox test. For the locomotion analysis (body bends per second and speed at day 7), around 150 tracks have been analyzed per group. Exact n numbers are indicated in data source. Data represent mean ± SEM from 3 independent repeats. The sample number for the pharyngeal pumping rates at day 4: n= 36 (control), n=30 (*pgph-2 o/e1*), n= 31 (*pgph-2 o/e4*), n=30 (*pgph-2 o/e6*), n=30 (control, *pgph-2 o/e1*, *pgph-2o/e4*, *pgph-2 o/6* on 2% glucose). P values are obtained using one-way ANOVA with Bonferroni correction. ****P<0.0001.
