# Supplementary material for: Phosphoglycolate phosphatase homologs act as glycerol-3-phosphate phosphatase to control stress and healthspan in C. elegans
Source: Nat Commun. 2022 Jan 11;13:177. doi: 10.1038/s41467-021-27803-6 (PMC8752807; doi:10.1038/s41467-021-27803-6)
Supplement: Supplementary file 2 — Description of Additional Supplementary Files [file 41467_2021_27803_MOESM2_ESM.docx]

**Description of Additional Supplementary Information**

**Phosphoglycolate phosphatase homologs act as glycerol-3-phosphate phosphatase to control stress and healthspan in *C. elegans***

Possik et al.,

**Supplementary Movies**

**File name: Supplementary Movie 1**

Description: Representative video showing the locomotion behavior of day 9 control animals on NGM plates.

**File name: Supplementary Movie 2**

Description: Representative video showing the locomotion behavior of day 9 *pgph-2 oe1* animals on NGM plates.

**File name: Supplementary Movie 3**

Description: Representative video showing the locomotion behavior of day 9 *pgph-2 oe4* animals on NGM plates.

**File name: Supplementary Movie 4**

Description: Representative video showing the locomotion behavior of day 9 *pgph-2 oe6* animals on NGM plates.

**File name: Supplementary Movie 5**

Description: Representative video showing the locomotion behavior of day 9 control animals on 2% glucose plates.

**File name: Supplementary Movie 6**

Description: Representative video showing the locomotion behavior of day 9 *pgph-2 oe1* animals on 2% glucose plates.

**File name: Supplementary Movie 7**

Description: Representative video showing the locomotion behavior of day 9 *pgph-2 oe4* animals on 2% glucose plates.

**File name: Supplementary Movie 8**

Description: Representative video showing the locomotion behavior of day 9 *pgph-2 oe6* animals on 2% glucose plates.

**Supplementary Data**

**File name: Supplementary Data 1**

Description: Hyperosmotic stress resistance assays: summary results and statistical analysis.

**File name: Supplementary Data 2**

Description: Lifespan assays: summary results and statistical analysis.

**File name: Supplementary Data 3**

Description: Glucotoxicity assays: summary results and statistical analysis.

**File name: Supplementary Data 4**

Description: Percent survival to oxidative stress (100 mM PQ): results and statistical analysis.

**File name: Supplementary Data 5**

Description: Percent recovery following 24 h anoxic injury: results and statistical analysis.

**File name: Supplementary Data 6**

Description: Percent recovery following 16 h cold stress: results and statistical analysis.

**File name: Supplementary Data 7**

Description: Transcription factor RNAi screen summary results.

**File name: Supplementary Data 8**

Description: Primers sequences.

**File name: Supplementary Data 9**

Description: Effect size calculations.
